# CD14^+^CD56^+^ Cell Is an Independent Regulatory Monocyte Subpopulation Increased in VKHS Patients Following Glucocorticoids Therapy

**DOI:** 10.1167/iovs.67.8.53

**Published:** 2026-07-24

**Authors:** Zixuan Wang, Han Jiang, Haiyu Deng, Fiona Huang, Shen Qu, Shan He, Bing Yu, Ting Ye, Zhaohui Li

**Affiliations:** 1 Aier Eye Hospital Group Co., Ltd.; Retinal and Vitreous Diseases Department of Wuhan Aier Eye Hospital, Wuhan University, Wuhan, People's Republic of China; 2 Shenzhen Baoan AirSea Hospital, Shenzhen, People's Republic of China; 3 Department of Pathogen Biology, School of Basic Medicine, Tongji Medical College, Huazhong University of Science and Technology, Wuhan, People's Republic of China; 4 Center for Reproductive Medicine, Department of Obstetrics and Gynecology, Peking University Third Hospital, Beijing, People's Republic of China; 5 Eye Center, Renmin Hospital of Wuhan University, Wuhan, People's Republic of China; 6 Wuhan Britain-China School, Wuhan, People's Republic of China; 7 Cancer Center, Union Hospital, Tongji Medical College, Huazhong University of Science and Technology, Wuhan, People's Republic of China

**Keywords:** glucocorticoids (GCs), autoimmune disease, Vogt-Koyanagi-Harada syndrome (VKHS), CD56^+^ monocyte, CD4^+^ T cells

## Abstract

**Purpose:**

Vogt-Koyanagi-Harada syndrome (VKHS) has been established as an autoimmune disease targeting melanocytes; however, its underlying mechanism remains obscure. We previously found an increased proportion of CD14^+^CD56^+^ monocyte (CD56^+^ monocyte thereafter) accompanied by decreased T cell frequency in the peripheral blood of patients with VKHS treated with glucocorticoids (GCs), suggesting the immunoregulatory function of this monocyte. However, whether CD56^+^ monocyte is a bona fide regulatory monocyte subset in VKHS is to be determined.

**Methods and Results:**

Through morphological, transcriptomic, and immunophenotypic analyses, we confirm that CD56^+^ monocyte constitutes a distinct immunoregulatory monocyte subset compared with CD56^−^ monocyte and NK cell. Upon lipopolysaccharide (LPS) stimulation, CD56^+^ monocytes showed greater capacity to secrete pro-inflammatory cytokines (TNF-α, IL-23, IL-8, and IL-6) and anti-inflammatory cytokine IL-10 compared with CD56^−^ monocytes and NK cells. Interestingly, secretion of granzyme B and perforin is observed in CD56^+^ monocytes but not CD56^−^ monocytes in steady-state conditions, albeit at lower levels than NK cells. Moreover, CD56^+^ monocytes demonstrate superior phagocytosis, antigen processing, and migration abilities but intermediate adhesion capabilities compared with CD56^−^ monocytes and NK cells. Importantly, monocytes treated in vitro and derived from GC-treated patients with VKHS harbor an increased proportion of CD56^+^ monocytes, and exhibit enhanced migration and reduced adhesion, along with stronger capacity to inhibit CD4^+^ T cell proliferation.

**Conclusions:**

These findings highlight the importance of CD56^+^ monocytes, as an independent regulatory monocyte subset, in response to GC therapy in the context of acute VKHS. The potential mechanism revealed in this study will provide insight into novel treatment strategies of VKHS by harnessing CD56^+^ monocytes.

Vogt-Koyanagi-Harada syndrome (VKHS) is a rare multisystem disease that characteristically affects various tissues containing melanocytes, including eyes, inner ear, meninges, and skin.^1^ Although the exact cause of VKHS remains unknown, the most accepted mechanism involves an autoimmune attack on antigens associated with melanocytes in genetically susceptible individuals following viral infection.^2^ In support of this notion, a crucial role of genetic factors in the pathogenesis of VKHS has been suggested by the higher susceptibility of individuals with *HLA-DR4/DRw53* alleles to this disease in China, North America, and Japan,^3^^–^^5^ whereas the effect of virus infection was indicated by the presence of cross-reactive T cells against tyrosinase following CMV infection.^6^ Moreover, patients carrying the HLA-DRB1*0405 allele harbor T cells that recognize a broader repertoire of melanocyte-derived peptides compared with HLA-matched control subjects.^7^ Additionally, in vitro experiments have shown the presence of cytotoxic CD4^+^ and CD8^+^ T lymphocytes in the peripheral blood of patients with VKHS targeting melanoma cells,^8^ suggesting the antigen-specific attack by autoreactive T cells against melanocytes.

The prerequisite for autoreactive T cells to perform their effector function is the activation of these cells induced by antigen-presenting cells (APCs), which function through presenting peptide antigens in the context of MHC molecules, and providing co-stimulatory signals as well as certain cytokines to T cells. Therefore, it is not surprising that major APCs, including dendritic cells, macrophages, and monocytes, have also been suggested to contribute to VKHS. Indeed, previous investigation from our group found that peripheral blood monocytes are significantly increased in patients with VKHS compared with healthy controls,^9^ suggesting their involvement in this autoimmune disease. Monocytes can exert their physiological or pathogenic functions in both peripheral blood and diverse tissues under steady-state conditions and during inflammation. Under steady-state conditions, monocytes perform vascular patrolling to maintain vessel integrity and migrate to tissues upon detecting abnormalities^10^^,^^11^; while during inflammation, monocytes are among the first immune cells to reach inflamed sites, where they mediate innate immune responses by recognizing, phagocytosing, degrading pathogens, and releasing pro-inflammatory cytokines.^12^^–^^15^ Monocytes can also present antigens directly, or indirectly after differentiating into macrophages and dendritic cells, thereby initiating and regulating adaptive immune responses. Recent studies indicate that monocytes are a heterogeneous population, comprising multiple subpopulations with distinct functions, among which a new subset, CD56^+^ monocyte, has been implicated in active Crohn’s disease and rheumatoid arthritis (RA).^16^^–^^18^ Interestingly, we found that the proportion and number of CD56^+^ monocytes are significantly higher in patients with acute VKHS than in healthy subjects, and further increased simultaneously with decreased frequency of T cell after glucocorticoid (GC) therapy,^9^ indicating their fluctuation during VKHS development and GC therapy. However, the precise role of CD56^+^ monocyte in VKHS and GC therapy remains to be determined.

Nevertheless, as the most common first-line therapeutic modality for VKHS,^1^ GC has already been reported to exert its anti-inflammatory effect through targeting monocytes and macrophages in addition to inhibiting autoreactive T and B cells.^19^^–^^21^ On one hand, GCs suppress innate inflammatory responses mediated by monocytes/macrophages: GCs inhibit the expression of pro-inflammatory cytokines (such as IL-1β, IL-6, IL-12, TNF, and GM-CSF) at the transcriptional level.^21^ Additionally, GC is also capable of inhibiting the synthesis of inflammatory mediators by suppressing enzyme production,^22^^,^^23^ and inducing the expression of anti-inflammatory mediators like IL-10 and CD163.^24^^,^^25^ Alternatively, GC treatment alters the cytoskeleton of human monocytes, reducing their adhesion to plastic surfaces and enhancing their spontaneous migration as well as migration toward fMLP, which may promote the differentiation of monocyte into anti-inflammatory subtypes followed by migrating to inflamed tissues, and thereby results in the resolution of inflammation.^26^^,^^27^ On the other hand, GCs indirectly regulate adaptive immune responses by modulating monocyte/macrophage functions: GC treatment reduces the expression of antigen-presenting molecules like CD80 on human monocytes, thereby decreasing their ability to activate effector T cells.^28^ In line with this, GC-treated mouse monocytes inhibit the proliferation of CD4^+^ and CD8^+^ T cells in vitro and suppress CD4^+^ T-cell-induced colitis in therapeutic settings.^29^ However, although we previously observed that the abundance of CD56^+^ monocytes is increased in response to GC therapy in patients with VKHS, whether these cells represent a GC-induced monocyte subset with unique regulatory capacity is still unclear. Thus, further studies are required to address this issue and elucidate the underlying mechanism.

To this end, this study started by performing a comprehensive comparison of CD56^+^ monocyte with CD56^−^ monocyte and NK cell at morphological, transcriptomic, and immunophenotypic levels to verify its identity as a unique cell population. Subsequently, we used in vitro experiments utilizing dexamethasone (DEX) to stimulate monocyte combined with in vivo examinations of monocyte responses to GC treatment to elucidate the mechanisms by which monocytes mediate the response of patients with VKHS to GC therapy. This study will provide evidence to clarify the role of monocytes in the natural course of VKHS and GC treatment, and help to explore GC therapeutic targets.

## Materials and Methods

### Study Subjects

In this study, patients with VKHS were recruited from the Retinal and Vitreous Diseases Department of Wuhan Aier Eye Hospital in January 2024. The diagnosis of the patient was based on the international uveitis meeting general diagnostic criteria for clinical diagnosis. Peripheral blood samples were collected from a patient with VKHS before and after GC treatment for 7 days. All healthy volunteers were recruited between April 10, 2022, and April 30, 2024, and their peripheral blood was simultaneously collected. This study was conducted in accordance with the Declaration of Helsinki, and approved by the Ethics Committee of Wuhan Aier Eye Hospital (Protocol Code: 2022IRBKY032501; Date of Approval: March 25, 2022). Informed consent was obtained from all individual participants included in the study.

### Reagents and Antibodies

The antibodies against CD14-AF647 (#325612), CD14-Biotin (#301826), CD56-APCcy7 (#318332), CD56-Biotin (#318320), CD86-PE (#305405), CD64-FITC (#305005), CD163-FITC (#333617), CX3CR1-FITC (#341605), NKp46-PEcy7 (#562101), CD69-APC (#310909), CD40L-PerCP/Cyanine5.5 (#310834), IL-8-AF488 (#511411), IL-10-PE (#501403), IL-1b-AF647 (#508207), IL-12/23-APC (#501809), IL-6-FITC (#501103), TNFα-PE (#376204), Streptavidin-PE (#405203), Streptavidin-AF488 (#405235), and human CD4 naïve T cell isolation kit (#480041) were purchased from Biolegend (San Diego, CA, USA). Fixable viability stain 510 (#564406), Fixable viability stain 700 (#564997), Fixation/permeabilization kit (#554714), and the antibodies against CD14-PE (#555398), CD56-BV421 (#562751), CD4-BB515 (#564419), CD4-BV510 (#562970), CD33-BV421 (#562854), CD11b-FITC (#557396), CD80-BV421 (#564160), HLADR-APC (#560744), CCR5-PEcy5 (#561750), CD54-BB515 (#564685), CD25-BV421 (#562442), CD134-FITC (#555837), CD137-PE (#555956), GZMB-PE (#561142), IFNy-BV421 (#562988), and IL-4-APC (#17-7049-42) were purchased from BD Biosciences (San Jose, CA, USA). The antibody against perforin-FITC (#1199442), Lysotracker (L7528), CellTrace Violet (C34557), FluoSpheres (F8803), and DQ-OVA (fluorescence-quenched ovalbumin substrate, D12053) were purchased from Invitrogen (Thermo Fisher Scientific, USA). Anti-PE microbeads (#130-048-801) were purchased from Miltenyi Biotec (Bergisch Gladbach, Germany). Mitotracker (C1048) was purchased from Beyotime Biotechnology (Shanghai, China). The reverse transcription kit (AG11728) and quantitative PCR (qPCR) kit (AG11701) were purchased from Aikerui Bioengineering (Changsha, China). The RNAprep pure micro kit (DP420) was purchased from TIANGEN Biotech (Beijing, China). Dex (HY14648) was purchased from MedChemExpress LLC (Monmouth Junction, NJ, USA). Lipopolysaccharide (LPS; L2630) was purchased from Sigma-Aldrich (St. Louis, MO, USA). DAPI staining solution (G1012) was purchased from Servicebio Technology (Wuhan, China).

### Collection and Isolation of Peripheral Blood Samples From Patients With VKHS and Healthy Individuals

Peripheral blood from the subjects was collected in heparinized tubes (5–30 mL) and transported at room temperature for processing. Then, a 500 µL sample was used for flow cytometric staining of monocyte surface markers, and the rest was diluted 1:1 with PBS to isolate peripheral blood mononuclear cells (PBMCs) using Ficoll density gradient centrifugation. Each 5 mL of blood mixed with 5 mL PBS was layered over 5 mL Ficoll in centrifuge tubes. After centrifuging at 800×*g* for 30 minutes, the buffy coat containing PBMCs was aspirated and washed with PBS. Then, the PBMCs were resuspended in 1 mL PBS, diluted, and mixed with trypan blue for cell counting.

### Flow Cytometry Staining

For whole blood staining, add 50 µL of fresh whole blood to a tube with Fc receptor blocking antibody, mix, and incubate at 4°C for 15 minutes. Then, add 50 µL of antibody solutions (prepared by adding appropriate amounts of antibodies against CD14, CD56, CD11b, HLA-DR, CD80, CD86, CD64, CD163, CX3CR1, or CCR5 into pre-cooled staining buffer), mix and incubate at 4°C in the dark for 30 minutes. Wash twice with 2 mL staining buffer (centrifuge at 500×*g* for 5 minutes). Lyse red blood cells with 2 mL lysis buffer, incubate for 10 minutes, wash again, and resuspend in 200 µL staining buffer with 1% paraformaldehyde for analysis.

As for surface staining of PBMCs, 1 × 10^6^ PBMCs per tube were blocked with Fc receptor blocking antibody followed by mixing and incubating at 4°C for 15 minutes. Subsequently, 30 µL of antibody solutions (prepared by adding appropriate amounts of antibodies against CD14, CD56, CD25, CD134, CD137, CD69, or CD40L and fixable viability staining [FVS] dye into pre-cooled staining buffer) were added, followed by mixing and incubating at 4°C in the dark for 30 minutes. Wash twice with 2 mL staining buffer (centrifuge at 500×*g* for 5 minutes), and resuspend in 200 µL staining buffer with 1% paraformaldehyde for analysis.

As for intracellular staining of granzyme B and perforin, after surface staining (above FVS dye), add 250 µL fixation/permeabilization solution, incubate at room temperature for 25 minutes. Wash with 1 mL PermWash solution, add intracellular antibody mixture (containing antibodies against granzyme B and perforin), incubate at 4°C for 30 minutes, wash again, and resuspend in 200 µL staining buffer with 1% paraformaldehyde for analysis.

As for intracellular staining of IL-1β, IL-8, IL-6, TNF-α, IL-23, and IL-10, an additional 5-hour stimulation of LPS was applied before fixation/permeabilization, and indicated antibody mixture are used instead of anti-granzyme B and perforin. The rest of the steps are identical to that used for staining of granzyme B and perforin.

### Sorting of Total Monocytes, CD56^+^ Monocytes, CD56^−^ Monocytes, and NK Cells

To purify total monocytes, anti-CD14-PE antibody was added to PBMCs solution after blocking with Fc receptor blocking antibody, and were then incubated for 30 minutes, washed, and added with anti-PE MicroBeads, followed by another round of incubation and wash. Subsequently, cells were resuspended in sorting-specific buffer and passed through an MS column on a magnetic stand to isolate CD14^+^ cells. The column was then rinsed three times and cells were eluted with buffer into a new tube. After centrifugation, the collected cells represent total monocytes. To purify CD56^+^ and CD56^−^ cells, anti-CD56-BV421 antibody was added to both CD14^+^ and CD14^−^ cell components, incubated for 30 minutes, washed, and then sorted using a flow cytometer into CD14^+^CD56^+^, CD14^+^CD56^−^, and CD14^−^CD56^+^ cells, representing CD56^+^ monocytes, CD56^−^ monocytes, and NK cells, respectively. Purified cells were used for RT-PCR, confocal microscopy, and in vitro experiments. For Smart-seq2, PBMCs were co-incubated with anti-CD14-PE and anti-CD56-BV421 antibodies, and sorted using a flow cytometer to ensure optimal cell viability.

### Detection of *CD14* and *NCAM1* Gene Expression in Monocytes and NK Cells

RNA of sorted CD56^+^ cells, CD56^−^ cells, and NK cells was extracted using a microRNA extraction kit (Tiangen, Shanghai, China). Reverse transcription was then performed to obtain cDNA, followed by qPCR analysis of the *CD14* and *NCAM1* (encoding CD56) genes using a qPCR instrument.

### Confocal Microscopy Observation of Organelle Structures in Monocytes and NK Cells

The sorted CD14^+^CD56^+^, CD14^+^CD56^−^, and CD14^−^CD56^+^ cells were resuspended in RPMI 1640 medium. Mitotracker was then added followed by incubating at 37°C for 30 minutes in the dark. After washing, biotin blocking agent was added and incubated at 4°C for 15 minutes. Subsequently, anti-CD56-biotin antibody was added and incubated at 4°C for 30 minutes. After washing, streptavidin-PE and anti-CD14-AF647 antibodies were then added and incubated for another 30 minutes. Cells were resuspended, and 50 µL was transferred to a confocal dish for observation. For revealing lysosomal morphology, lysotracker was used, and streptavidin-PE in the above procedure was replaced with streptavidin-AF488.

### Smart-seq2

Then, 1 × 10^7^ PBMCs were sorted into 200 µL centrifuge tubes containing lysis buffer using a flow cytometer, ensuring 50 cells each of CD14^+^CD56^+^, CD14^+^CD56^−^, and CD14^−^CD56^+^. Tubes were briefly centrifuged, flash-frozen in liquid nitrogen, and stored at −80°C for RNA extraction. Sequencing was performed on an Illumina NovaSeq 6000 with PE150 reads. STAR aligned reads to the reference genome, and gene counts were normalized using transcripts per million (TPM) for principal component analysis (PCA), clustering, and correlation analyses. Deseq2 identified differentially expressed genes (determined by |log2(FoldChange)| ≥ 1 and adjusted *P* [adj *P* value] ≤ 0.05), followed by Gene Ontology (GO) and Kyoto Encyclopedia of Genes and Genomes (KEGG) pathway enrichment analysis. Raw sequencing data generated in this study were deposited in the Science Data Bank under the accession number (DOI: 10.57760/sciencedb.29177).

### Phagocytosis Assay of Monocytes

Then, 1 × 10^6^ PBMCs were incubated with FITC-labeled latex beads at 37°C for 4 hours, and were then stained with anti-CD14, anti-CD56 antibodies and FVS dye. The proportions of CD14^+^CD56^+^, CD14^+^CD56^−^, and CD14^−^CD56^+^ cells containing beads were analyzed by flow cytometry.

### Antigen Processing Assay of Monocytes and NK Cells

Then, 1 × 10^6^ PBMCs were incubated with 70 µg/mL DQ-OVA at 37°C for 5 hours. After washing, cells were stained with anti-CD14, anti-CD56 antibodies, and FVS dye at 4°C for 30 minutes. The uptake and processing of DQ-OVA by CD14^+^CD56^+^, CD14^+^CD56^−^, and CD14^−^CD56^+^ cells were analyzed using a flow cytometer.

### Migration Assay of Monocytes and NK Cells

CD14^+^ monocytes were isolated using magnetic beads, and CD14^+^CD56^+^, CD14^+^CD56^−^, and CD14^−^CD56^+^ cells were purified by combining magnetic bead separation with flow cytometric sorting. Cells were resuspended in RPMI 1640 without FBS. Each group (2 × 10^4^ cells) was placed in a Transwell insert, and migration to the lower chamber containing 20% FBS was assessed after 4 hours at 37°C. Migration rates were compared by counting cells in the lower chamber.

### Adhesion Assay of Monocytes and NK Cells

Cells were resuspended in RPMI 1640 without FBS and incubated at 37°C for 4 hours. After fixation with 4% PFA and PBS washes, DAPI staining was applied. Cells were counted using a plate scanner (total monocytes) or fluorescence microscope (five random fields for subpopulations). The adhesion rate was determined by comparing the average number of cells per field of view.

### Co-Culture Experiment of Monocytes and Naïve CD4^+^ T Cells

Monocytes isolated using magnetic beads were stimulated with 10^−6^ M DEX for 5 hours at 37°C. Naïve CD4^+^ T cells were obtained from CD14^−^ cells via negative selection using a biotinylated antibody cocktail and streptavidin-conjugated nanobeads, followed by incubation on ice and separation using a MojoSort magnet. For proliferation detection, naïve CD4^+^ T cells were stained with CellTrace Violet (CTV) in PBS, incubated at 37°C for 30 minutes, and terminated with FBS. Cells were then washed and resuspended in staining buffer. Monocytes and naïve CD4^+^ T cells were then co-cultured at a 1:1 ratio in 96-well plates at 37°C for 72 hours. After incubation, cells were collected and stained for surface markers using flow cytometry.

### Statistical Methods and Data Analysis Software

Flow cytometry data were analyzed using FlowJo V10 and visualized with R packages. Statistical analyses were performed with GraphPad Prism 8.0, using *t*-tests for two datasets and ANOVA with Dunnett’s test for three or more. *P* < 0.05 was determined as significant, denoted by *, **, ***, and **** for *P* < 0.05, 0.01, 0.001, and 0.0001, respectively.

## Results

### In Vitro Dexamethasone Stimulation of PBMCs and Monocytes Increases the Proportion of CD56^+^ Monocytes, Mimicking the Findings in Patients With VKHS After GC Treatment

**Figure 1.**
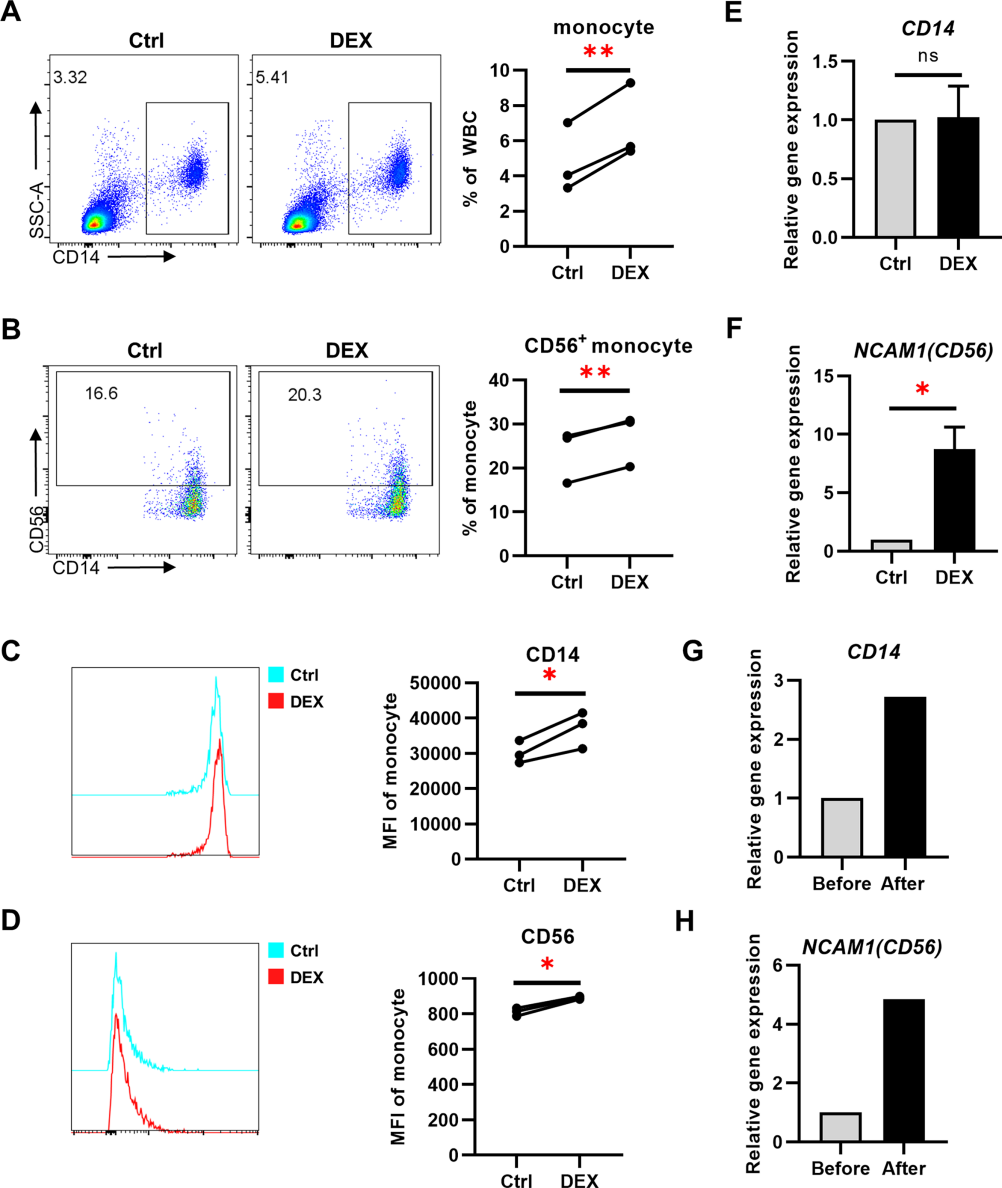
**GC treatment increases CD56^+^ monocyte proportion.** (**A–F**) After stimulating PBMCs with 10^−6^ M DEX for 5 hours, the changes in total monocytes and CD56^+^ monocytes were compared with the control group (Ctrl) (*n* = 3). Representative dot plots and bar graphs with statistical analyses showing the proportion change of total monocytes **A** and CD56^+^ monocytes **B**. Representative histograms and bar graphs with statistical analyses showing MFI of CD14 in total monocytes **C**, and of CD56 in total monocytes **D**. Bar graphs showing the relative expression of *CD14* in total monocytes **E**, and of *NCAM1* in total monocytes **F**, as determined by real-time RT-PCR. (**G,** **H**) Bar graphs showing the relative expression levels of *CD14* **G** and *NCAM1* **H** in total monocytes from a patient with VKHS before and after GC therapy, as determined by real-time RT-PCR. MFI, mean fluorescence intensity; *P < 0.05; NS, not significant. A *t*-test was used to analyze the two datasets.

We previously found that compared with healthy controls, patients with VKHS have increased total peripheral monocytes, particularly CD56^+^ monocytes. Interestingly, GC treatment further increased the proportion of CD56^+^ monocytes,^9^ suggesting their role in VKHS and GC therapy. Based on these observations, we asked if in vitro treatment of PBMC with GC could also increase the proportion of CD56^+^ monocytes. To test this, PBMCs were isolated from healthy individuals and treated with DEX for 5 hours, followed by flow cytometric staining with antibodies for CD14 and CD56, to examine the changes in the proportions of total monocytes and CD56^+^ monocytes after DEX stimulation. Our results showed that compared with the control group, the proportion of total monocytes in PBMCs significantly increased after in vitro GC stimulation (Fig. 1A), and the proportion of CD56^+^ monocytes in the DEX group increased even more markedly (Fig. 1B). Mean fluorescence intensity (MFI) analysis of CD14 and CD56 also showed significant increases in the DEX-treated group (Figs. 1C, 1D). These results collectively indicate that DEX stimulation in vitro can recapitulate the changes in monocytes observed in vivo following GC therapy.

However, GC treatment of PBMC may induce the above changes in monocytes indirectly by acting on other immune cells. Additionally, false positive cells resulted from the nonspecific binding of antibodies against CD14 and/or CD56 to other cells, and the formation of monocyte-other cell doublets due to the increased adhesion of monocytes after in vitro culture, can both lead to the overestimation of our above results in flow cytometric analyses. To exclude these possibilities, and to confirm the direct effect of GC treatment on monocytes, we used magnetic bead separation to isolate monocytes and exclude doublets before DEX stimulation, and used RT-PCR to determine the transcriptional expression of CD14 and CD56. The results showed no significant difference in CD14 gene expression but significantly higher *NCAM1* (encoding CD56) expression in the DEX group (Figs. 1E, 1F), which is consistent with changes observed in patients with VKHS after GC therapy^9^ (Figs. 1G, 1H). Therefore, these results demonstrate that in vitro DEX stimulation of PBMC/monocyte can mimic in vivo GC treatment of patients with VKHS in increasing the proportion of CD56^+^ monocytes.

### CD56^+^ Monocytes Represent an Independent Subset of Monocytes Differing From CD56^−^ Monocytes and NK Cells

CD56^+^ monocytes have been reported to be elevated in various autoimmune diseases, including active Crohn’s disease and rheumatoid arthritis.^17^^,^^18^ Furthermore, our previous findings suggest that these cells may also play a regulatory role in VKHS.^9^ However, whether CD56^+^ monocytes function as an independent monocyte subset still requires further validation. For this purpose, we preliminarily validated CD56^+^ monocytes as a distinct subset based on morphology and size using immunofluorescence technique (Supplementary Fig. S1A). To further strengthen this evidence, CD14^+^CD56^+^ (CD56^+^ monocytes), CD14^+^CD56^−^ (CD56^−^ monocytes), and CD14^−^CD56^+^ cells (NK cells) were purified by flow cytometry and compared for their differences in morphology, and CD14/CD56 expressions at transcriptional and protein levels.

**Figure 2.**
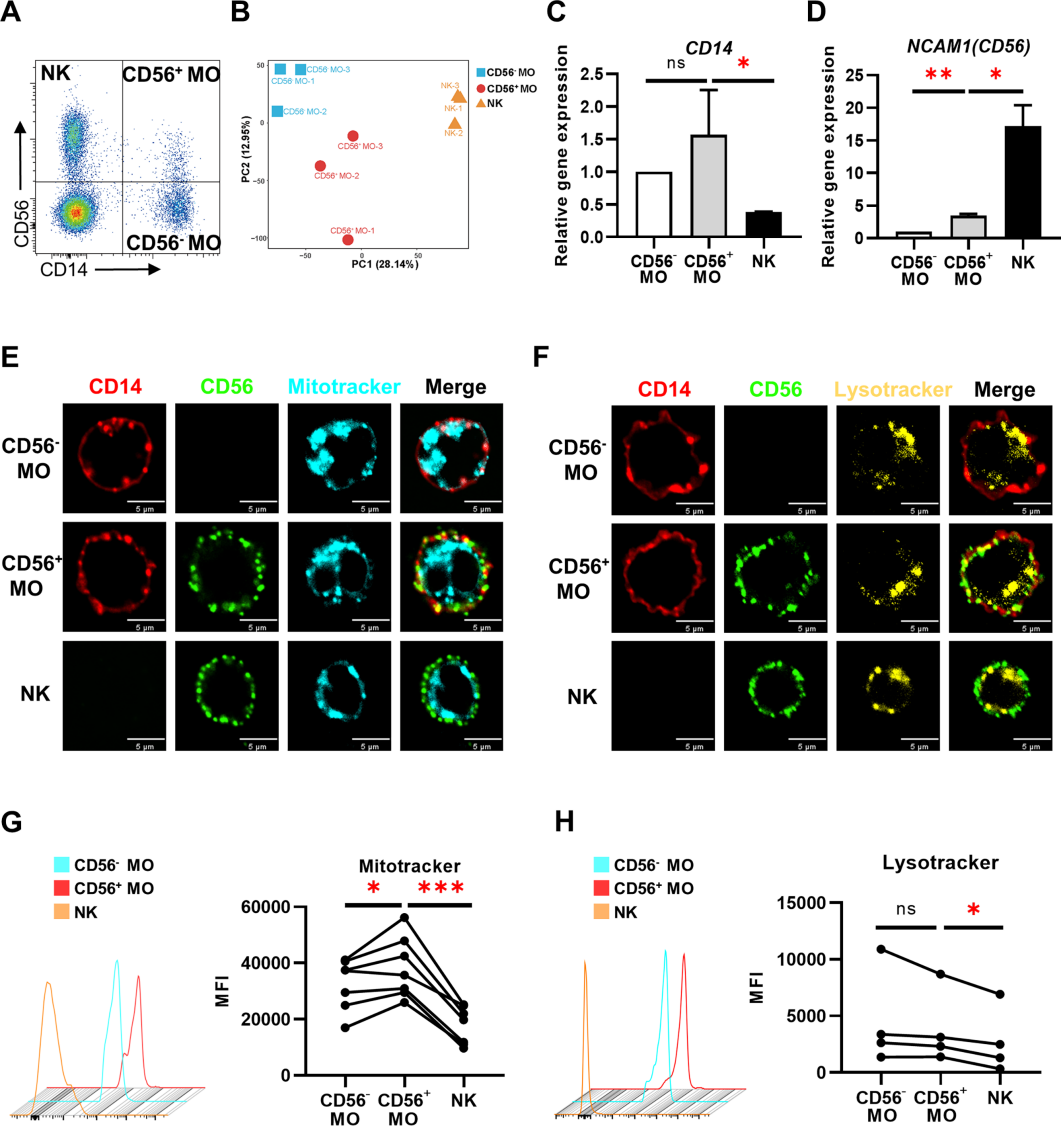
**CD56^+^ monocytes represent a distinct and independent monocyte subset with unique characteristics compared** **with** **CD56^–^** **monocytes and NK cells.** (**A**) Flow cytometry gating strategy of the three cell populations (*n* = 3). (**B**) Principal component analysis (PCA) plot based on RNA-sequencing data showing the separation of these three cell populations (*n* = 3). (**C**) Expression of the *CD14* (*n* = 3) analyzed by RT-PCR. (**D**) Expression of the *NCAM1* (*n* = 3) analyzed by RT-PCR. (**E**) Representative confocal laser microscopy images of mitochondria in CD56^+^ monocytes, CD56^−^ monocytes, and NK cells. CD14 is labeled in *red*, CD56 in *green*, and mitochondria (Mitotracker) in *yellow*. (**F**) Representative confocal laser microscopy images of lysosomes in CD56^+^ monocytes, CD56^−^ monocytes, and NK cells. CD14 is labeled in *red*, CD56 in *green*, and lysosomes (Lysotracker) in *yellow*. (**G**) Mitotracker MFI (*n* = 7) measured in CD56^+^ monocytes, CD56^−^ monocytes, and NK cells derived from PBMCs, as analyzed by flow cytometry. (**H**) Lysotracker MFI (*n* = 4) measured in CD56^+^ monocytes, CD56^−^ monocytes, and NK cells derived from PBMCs, as analyzed by flow cytometry. MO, monocytes; MFI, mean fluorescence intensity; ***P < 0.001; **P < 0.01; *P < 0.05; NS, not significant. Datasets with three groups were analyzed using 1-way ANOVA, followed by Dunnett’s multiple comparisons test.

As shown in the representative flow cytometric plot (Fig. 2A), the expressions of CD56 are high, moderate to low, and undetectable, in NK cells, CD56^+^ monocytes and CD56^−^ monocytes, respectively. When these cells were sorted and subject to RNA-sequencing followed by bioinformatics analyses, the separation of these cell populations was immediately evident, as demonstrated in PCA (Fig. 2B). RT-PCR confirmed the higher *CD14* expression in both monocyte subpopulations compared with NK cells (Fig. 2C). As expected, *NCAM1* (encoding CD56) expression was the highest in NK cells, and notably, was significantly higher in CD56^+^ monocytes compared to that in CD56^−^ monocytes (Fig. 2D). Therefore, these results further confirm the expression of CD56 in CD14^+^CD56^+^ cells at the transcriptional level. Moreover, confocal microscopy results showed that both CD14 and CD56 are co-expressed and colocalized on the surface of CD56^+^ monocytes, which is in line with our preliminary findings (see Supplementary Fig. S1A). In contrast, CD56^−^ monocytes expressed only CD14 on the cell surface, and NK cells did not express CD14 at all. Of note, CD56 expression on the cell membrane was discontinuous and punctate, whereas CD14 expression was continuous but with multiple breakpoints (Figs. 2E, 2F), indicating different expression patterns for the two molecules. Next, we used MitoTracker and LysoTracker to label mitochondria and lysosomes, respectively, showing that both CD56^+^ and CD56^−^ monocytes exhibited higher mitochondrial and lysosomal contents, than NK cells (see Figs. 2E, 2F). We next performed flow cytometric analysis to quantitively confirm the above findings. Indeed, the two monocyte subpopulations exhibited higher mitochondrial fluorescence intensity than NK cells, and, interestingly, higher intensity was noted in CD56^+^ monocytes compared with that in CD56^−^ monocytes (Figs. 2G, 2H). This finding is in concordance with the higher ROS content, which may impact mitochondrial function and oxidative stress,^30^ in CD56^+^ monocytes (Supplementary Fig. S2). As for lysosome, higher lysosome content in monocytes was observed compared with NK cells, albeit the difference between CD56^+^ and CD56^−^ monocytes was not statistically significant. In conclusion, whereas CD56^+^ monocytes share similarities with CD56^−^ monocytes in terms of morphology, CD14 expression, and lysosome content, which are distinct from NK cells and indicate their identity as bona fide monocytes, they exhibit markedly higher CD56 expression at both transcriptional and protein levels, and unique mitochondrial and lysosomal characteristics than CD56^−^ monocytes, supporting their classification as an independent monocyte subset. These features likely contribute to their distinct functions in immune regulation and autoimmune diseases, as we previously suggested.^9^

### CD56^+^ Monocytes Exhibit Characteristics of Both Monocytes and NK Cells at the Transcriptional Level

**Figure 3.**
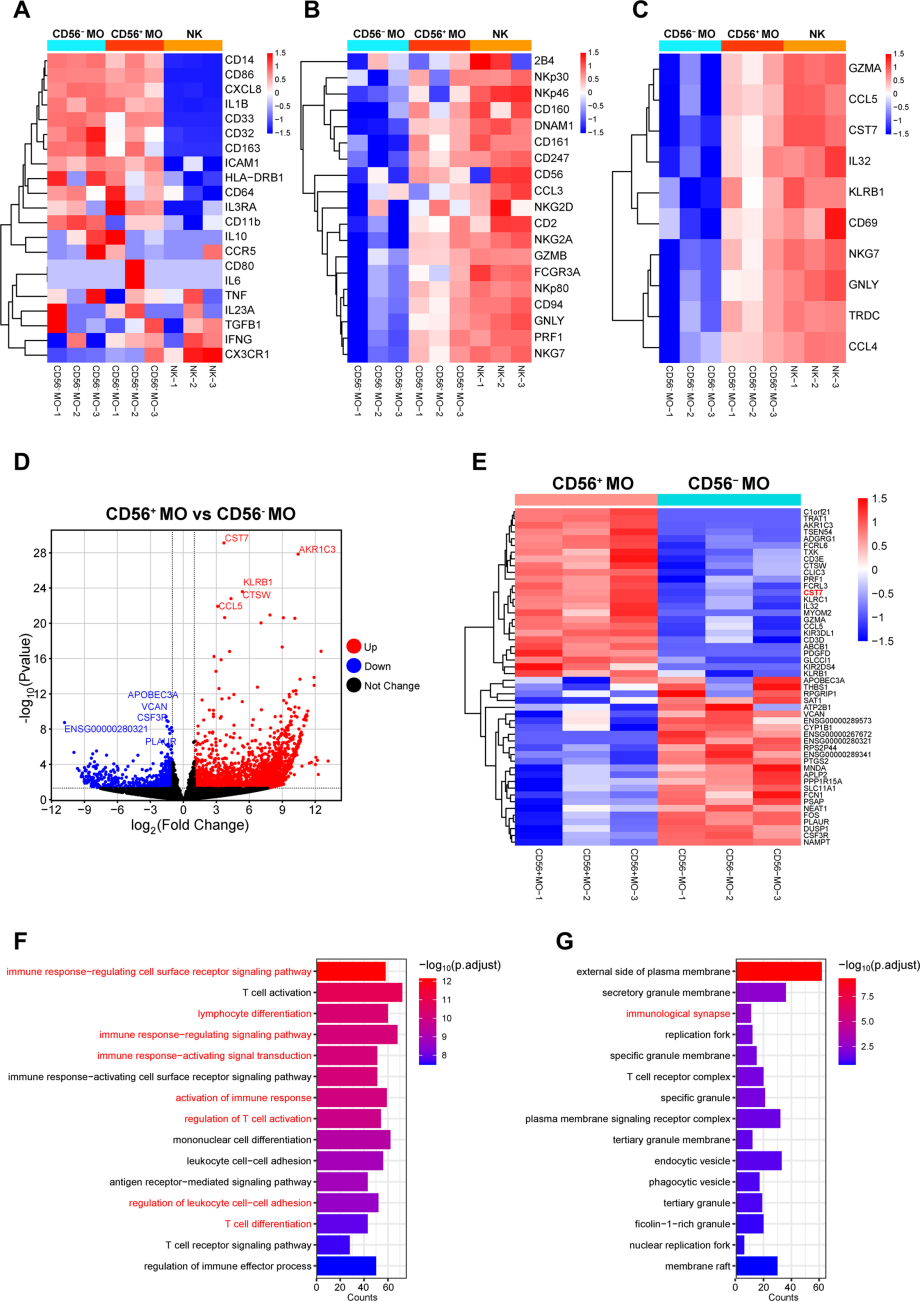
**CD56^+^ monocytes display transcriptional features shared with both monocytes and NK Cells.** (**A–C**) Comparison of characteristic gene expression levels among CD56^+^ monocytes, CD56^−^ monocytes, and NK cells after PBMC sorting. Heatmap plot showing the relative expressions of monocyte-characteristic genes **A**, NK-characteristic genes **B**, and NK-like monocyte-characteristic genes **C**. (**D,** **E**) Differential gene analysis between CD56^+^ monocytes and CD56^−^ monocytes. Volcano plot displaying differentially expressed genes. *Red dots* represent upregulated genes, *blue dots* represent downregulated genes, and *black dots* represent non-differentially expressed genes **D**. Heatmap showing the top 50 differentially expressed genes. *Red* indicates upregulated genes, and *blue* indicates downregulated genes **E**. (**F,** **G**) GO pathway enrichment analysis of CD56^+^ monocytes and CD56^−^ monocytes. GO analysis results for biological process **F**. GO analysis results for cellular component (CC) **G**.

Next, we used smart-seq2 sequencing followed by bioinformatic analyses to profile the transcriptional differences among CD56^+^ monocytes, CD56^−^ monocytes, and NK cells. As shown in the heatmap plot in Figure 3A, CD56^+^ monocytes express typical monocyte marker genes including *CD14*, *CD33*, *CD11b*, *CD86*, *CD163*, *ICAM1*, *CD32*, *CD64*, *HLA-DRB1*, *IL1B*, *CXCL8*, and *IL3RA*, confirming their monocyte identity. Interestingly, CD56^+^ monocytes exhibit higher expression of cytotoxicity-related genes such as *GZMB*, *PRF1*, *NKG2D*, *NKp30*, *NKp46*, *CST7*, and *FCGR3A*, compared with CD56^−^ monocytes, albeit at lower levels than NK cells (Figs. 3B, 3C). This result is consistent with the differential CD56 expression in these cells observed at both protein and transcriptional levels (see Figs. 2A, 2D), together suggesting their potential cytotoxic functions similar to NK cells. Recent single-cell sequencing data from patients with VKHS and healthy individuals identified a population of NK-like monocytes expressing signature genes such as *GZMA*, *CCL5*, *CST7*, *IL32*, *KLRB1*, *CD69*, *NKG7*, *GNLY*, *TRDC*, and *CCL4.*^31^ Interestingly, the expression levels of these signature genes in CD56^+^ monocytes are intermediate between that in CD56^−^ monocytes and NK cells (see Fig. 3C). Therefore, our transcriptional analyses indicate that CD56^+^ monocytes share gene expression characteristics with both monocytes and NK cells, suggesting that whereas CD56^+^ monocytes may possess functions typical of monocytes, such as phagocytosis, antigen processing, adhesion, and migration, they might also have cytotoxic functions or regulatory roles similar to those of NK cells.

Next, we performed differential gene expression analysis of CD56^+^ and CD56^−^ monocytes, resulted in the identification of 2965 differentially expressed genes, with 2284 upregulated and 681 downregulated in CD56^+^ monocytes (Fig. 3D). Again, cytotoxicity-related gene *CST7* was notably upregulated by approximately 12.9-fold in CD56^+^ monocytes (Fig. 3E), further suggesting the cytotoxic potential of these cells. Furthermore, GO analysis of biological process enriched pathways related to immune response activation, immune response regulation, regulation of T cell activation, regulation of leukocyte cell-cell adhesion, and lymphocyte differentiation in CD56^+^ monocytes (Fig. 3F). Similarly, GO analysis of cellular component also enriched pathways associated with immune receptor activation and immunological synapse (Supplementary Figs. S3A, S3G). In addition, KEGG pathway analysis further identified NK cell mediated cytotoxicity, Th1 and Th2 cell differentiation pathways as enriched in CD56^+^ monocytes (Supplementary Fig. S3B). Together, these findings suggest that CD56^+^ monocytes may play a role in immune regulation by modulating the proliferation and differentiation of CD4^+^ T cells.

### CD56^+^ Monocytes Show an Immune Phenotype Incorporating Features of Both Monocytes and NK Cells

**Figure 4.**
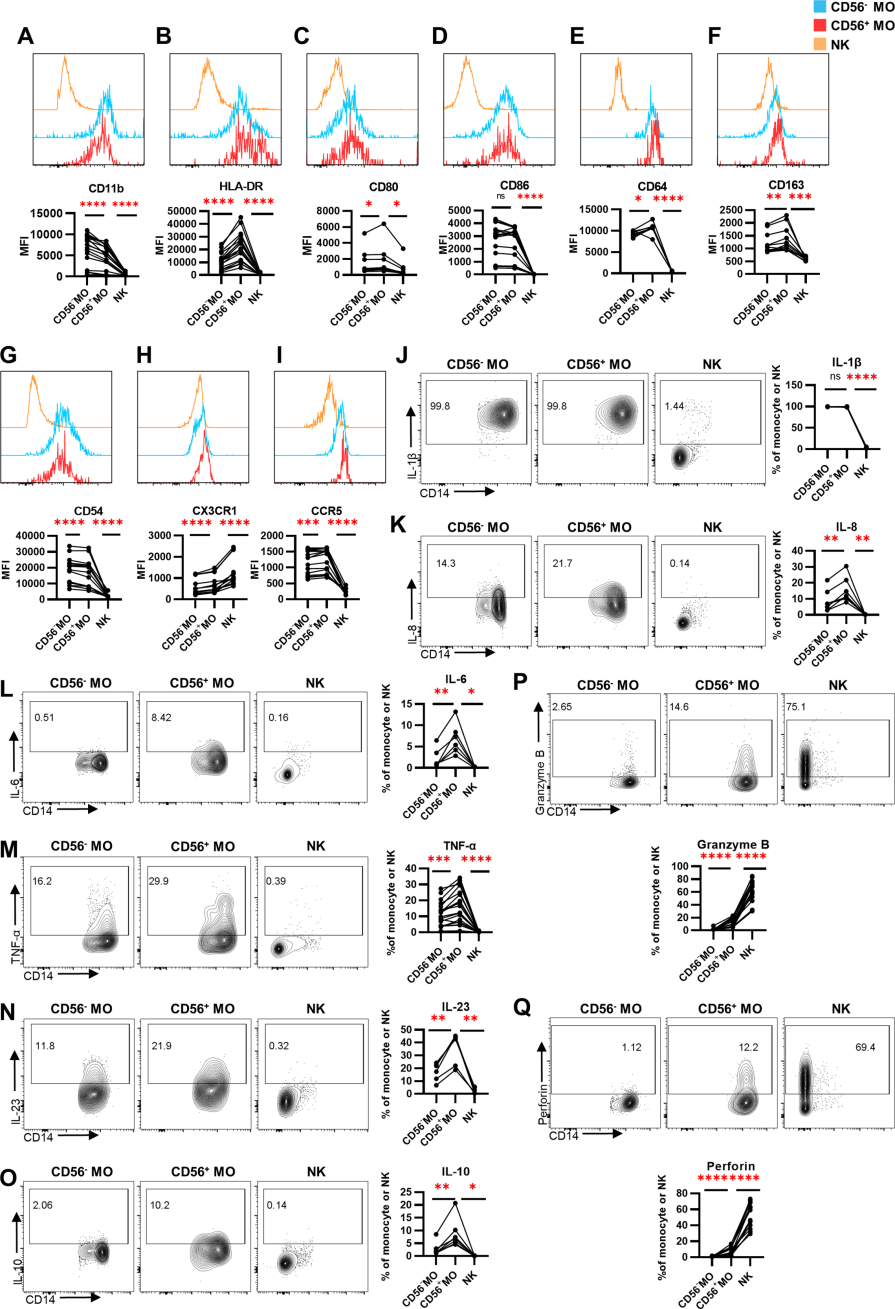
**CD56^+^ monocytes exhibit an immune phenotype characteristic of both monocytes and NK Cells.** (**A–I**) Flow cytometric analysis of MFI of surface markers related to activation, phagocytosis, adhesion, and chemotaxis in CD56^+^ monocytes, CD56^−^ monocytes, and NK cells. Histograms (*upper panels*) and line graphs with statistical analyses (*lower panels*) showing the MFI of CD11b (*n* = 18) **A**, HLA-DR (*n* = 18) **B**, CD80 (*n* = 13) **C**, CD86 (*n* = 16) **D**, CD64 (*n* = 9) **E**, CD163 (*n* = 12) **F**, CD54 (*n* = 16) **G**, CX3CR1 (*n* = 13) **H**, and CCR5 (*n* = 16) **I**. (**J–O**) CD56^+^ monocytes, CD56^−^ monocytes, and NK cells were stimulated with LPS, 5 hours later, intracellular staining of indicated cytokines and flow cytometric analyses were performed to evaluate the cytokine-secreting potential of these cells. Representative dot plots (*upper panels*) line graphs with statistical analyses (*lower panels*) showing the percentage of cells expressing IL-1β (*n* = 12) **J**, IL-8 (*n* = 7) **K**, IL-6 (*n* = 6) **L**, TNF-α (*n* = 20) **M**, IL-23 (*n* = 6) **N**, IL-10 (*n* = 7) **O**. (**P,** **Q**) CD56^+^ monocytes, CD56^−^ monocytes, and NK cells were directly examined by intracellular flow cytometry for their expression of cytotoxic molecules. Representative dot plots (*upper panels*) line graphs with statistical analyses (*lower panels*) showing the percentage of cells expressing granzyme B (*n* = 14) **P** and perforin (*n* = 14) **Q**. MFI, mean fluorescence intensity; MO, monocytes; ****P < 0.0001; ***P < 0.001; **P < 0.01; *P < 0.05; NS, not significant. Datasets with three groups were analyzed using 1-way ANOVA, followed by Dunnett’s multiple comparisons test.

Having characterized the transcriptional feature of CD56^+^ monocytes, we went on to compare the immune phenotype of these cells to those of CD56^−^ monocytes and NK cells using flow cytometry. We first analyzed surface markers typically found in monocytes, including CD11b (a pan-myeloid marker that plays an important role in cell adhesion, migration, and activation), CD54 (an adhesion molecule mediating cell-cell interaction), HLA-DR, CD80, and CD86 (three molecules mediating antigen presentation upon cell activation), Fc receptor CD64 and scavenger receptor CD163 that both participate in phagocytosis, and chemokine receptors CCR5 and CX3CR1 that guide the entrance of cells into different tissues. As expected, NK cells did not show expression in any of these markers, whereas two monocyte subsets exhibit differential expression levels. Specifically, unlike NK cells, CD56^+^ monocytes express the pan-myeloid marker CD11b, albeit lower than CD56^−^ monocytes (Fig. 4A). Interestingly, significantly higher expression of HLA-DR, CD80, CD64, and CD163 (but not CD86) were observed in CD56^+^ monocytes compared with CD56^−^ monocytes (Figs. 4B–F), suggesting the activated status and enhanced phagocytosis of these cells. Moreover, chemokine receptors CCR5 and CX3CR1 are markedly higher (Figs. 4H, 4I), whereas adhesion molecule CD54 was lower (Fig. 4G), in CD56^+^ monocytes compared with CD56^−^ monocytes. Thus, these results suggest that CD56^+^ monocytes may be a monocyte subpopulation with a higher degree of activation, and higher capacity of detaching from blood vessels and migrating to extravascular sites to exert their immune functions.

In addition to expressing membrane molecules, the functional activities of monocytes also depend on the secretion of cytokines.^12^ Cytokine secretion under LPS stimulation revealed that NK cells do not secrete typical pro-inflammatory or anti-inflammatory factors produced by monocytes. In contrast, upon LPS stimulation, both types of monocytes produce comparable high levels of IL-1β (Fig. 4J), and, interestingly, CD56^+^ monocytes secreted higher amounts of pro-inflammatory cytokines including IL-8, IL-6, TNF-α, and IL-23 (Figs. 4K–N), and the anti-inflammatory cytokine IL-10 (Fig. 4O). Therefore, CD56^+^ monocytes possess the potential to secrete both pro-inflammatory and anti-inflammatory cytokines, and this dual effect may reflect their role in maintaining immune balance.

NK cells, as classical killer cells, are known for their capacity to kill target cells through a well-known mechanism: when activating signals outweigh inhibitory signals, NK cells form immune synapses and release extracellular vesicles containing perforin and granzymes to destroy the target cells.^32^ Previous studies indicated that GC-stimulated monocytes can differentiate into tolerogenic dendritic cells (TolDCs)^33^^,^^34^ that are capable of secreting granzyme B and perforin.^35^^–^^37^ Given that unlike CD56^−^ monocytes, CD56^+^ monocytes express genes related to cytotoxic functions (see Figs. 3B–E), we went on to examine the protein expression levels of granzyme B and perforin, the two important cytotoxic molecules typically expressed in NK cells and CTLs, in CD56^+^ monocytes under steady state using flow cytometry. Our results showed that the expression of these molecules are neglectable in CD56^−^ monocytes (granzyme B = 1.88% ± 1.67 and perforin = 0.59% ± 0.51), whereas are significantly increased in CD56^+^ monocytes (granzyme B = 12.75% ± 6.61 and perforin = 8.51% ± 4.61), albeit lower than NK cells (granzyme B = 62.00% ± 17.26 and perforin = 51.66% ±16.71; Figs. 4P, 4Q). Thus, together with our transcriptomic results, these findings suggest that CD56^+^ monocytes likely possess some cytotoxic capabilities through these cytotoxic molecules, contributing to their potential roles in immune regulation and target cell killing.

### CD56^+^ Monocytes Exhibit Unique In Vitro Function Differing From CD56^−^ Monocytes and NK Cells

Our morphological, transcriptomic, and immunophenotypic analyses suggest that CD56^+^ monocytes may have distinct functions compared with CD56^−^ monocytes and NK cells. Therefore, we next conducted in vitro functional experiments to directly address this hypothesis.

**Figure 5.**
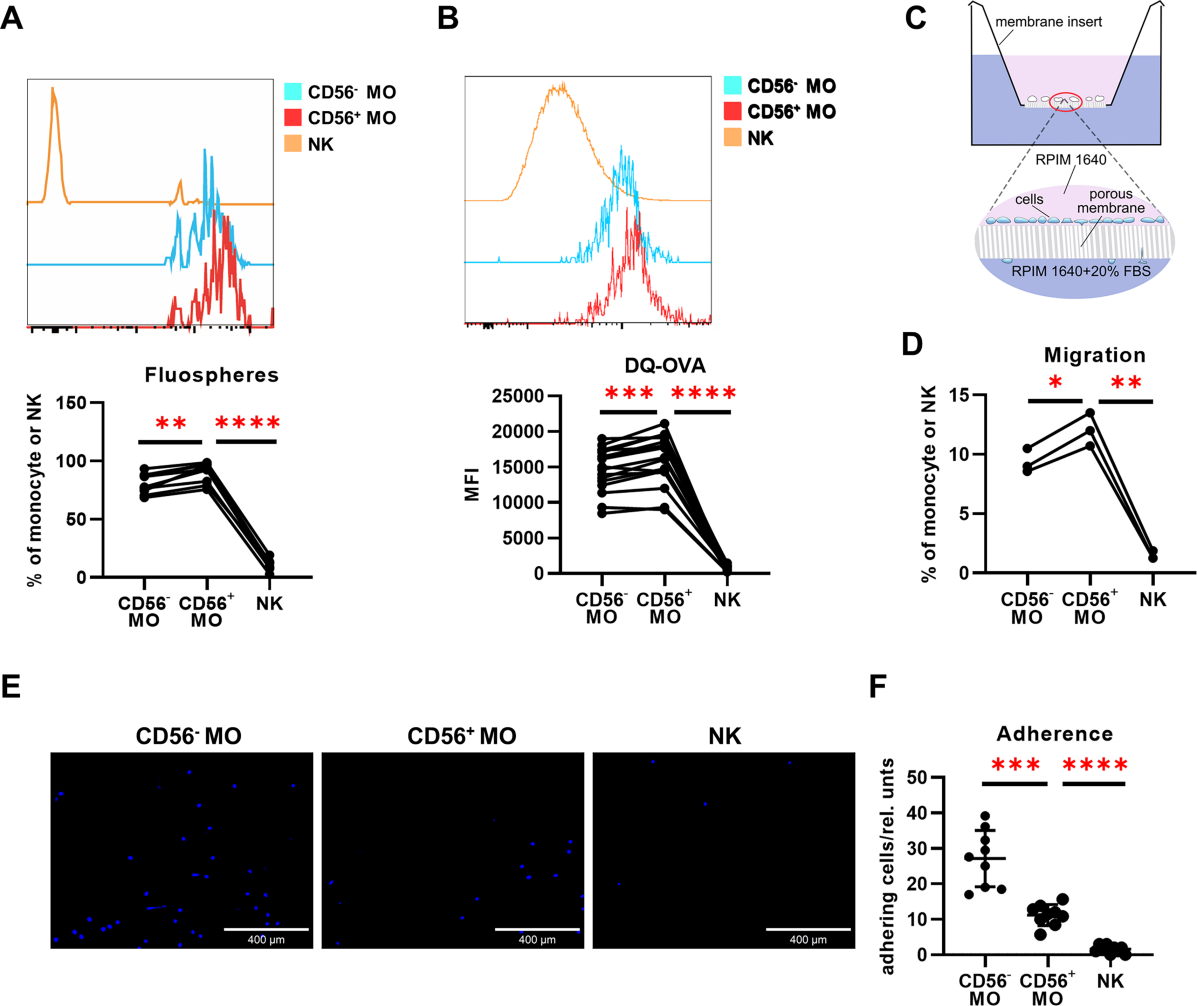
**CD56^+^ monocytes exhibit distinct in vitro functional properties compared to CD56** **^–^** **monocytes and NK cells.** (**A**) The proportions of CD14^+^CD56^+^ monocytes, CD14^+^CD56^−^ monocytes, and CD14^−^CD56^+^ NK cells containing latex microspheres were quantified by flow cytometry (*n* = 9). (**B**) The ability of CD14^+^CD56^+^ monocytes, CD14^+^CD56^−^ monocytes, and CD14^−^CD56^+^ NK cells to uptake and process the DQ-OVA model antigen (represented as green mean fluorescence intensity) using flow cytometry (*n* = 17). (**C**) Schematic diagram of the migration experiment. (**D**) The migration rates of CD56^+^ monocytes, CD56^−^ monocytes, and NK cells to 20% serum medium (*n* = 3). (**E**) DAPI fluorescence images of monocytes adhered to plastic plates in serum-free medium. (**F**) Numbers of adhered cells (*n* = 3). Each dot represents the average count from three fields of view at 200 × magnification, obtained from samples of three different healthy individuals. MFI, mean fluorescence intensity; MO, monocytes. ****P < 0.0001; ***P < 0.001; **P < 0.01; *P < 0.05. Datasets with three groups were analyzed using 1-way ANOVA, followed by Dunnett’s multiple comparisons test.

We first compared the phagocytic activity of CD56^+^ monocytes, CD56^−^ monocytes, and NK cells toward latex beads, showing that CD56^+^ monocytes had a significantly higher phagocytic capacity to engulf latex beads compared with CD56^−^ monocytes and NK cells (Fig. 5A). These results are in concordance with the higher levels of phagocytosis-related markers *CD64* and *CD163* in CD56^+^ monocytes revealed by gene expression analysis (see Fig. 3A), and a higher proportion of CD64^+^CD56^+^ and CD163^+^CD56^+^ cells demonstrated by flow cytometric analysis (see Figs. 4E, 4F), together indicating the enhanced phagocytic capacity of CD56^+^ monocytes compared with CD56^−^ monocytes.

Similarly, higher expression of antigen-presenting molecules HLA-DR and CD80 in CD56^+^ monocytes (see Figs. 4B, 4C) suggest that these cells may have enhanced antigen-presenting capacity. Indeed, DQ-OVA treatment experiments demonstrated that CD56^+^ monocytes had significantly greater antigen-processing capabilities, as indicated by the higher green fluorescence intensity (Fig. 5B).

Monocytes respond to an array of chemokines (e.g. CCL2, CCL3, CCL4, CCL5, and CX3CL1) released at inflamed sites. These chemokines bind to receptors on monocytes (e.g. CCR1, CCR2, CCR3, CCR5, and CX3CR1), triggering intracellular signaling pathways that direct monocytes to move toward inflamed or infected areas. Sequencing results showed increased expression of *CCR5* and *CX3CR1* in CD56^+^ monocytes (see Fig. 3A), which was consistent with protein expression levels (see Figs. 4H, 4I), together suggesting increased migratory potential in CD56^+^ monocytes. In support of this notion, Transwell migration assays confirmed that CD56^+^ monocytes had significantly higher migration capacities compared with CD56^−^ monocytes, whereas NK cells showed minimal response (Figs. 5C, 5D).

In addition to chemokine receptors, monocytes also express a number of adhesion molecules such as CD54, the dynamic expression of which has a major influence on the biological activities of monocytes including but not limit to detachment of monocytes from vascular wall, extravasating into tissues, and interaction with other cells, especially during inflammatory response.^12^ Sequencing and flow cytometric results showed lower CD54 expression in CD56^+^ monocytes (see Figs. 3A, 4G). Here, adhesion assays on a plastic surface revealed that although both CD56^+^ and CD56^−^ monocytes have adhering capacity, CD56^+^ monocytes exhibited significantly weaker adhesion (Figs. 5E, 5F), suggesting CD56^+^ monocytes might be more prone to detachment from the vasculature. Thus, despite weaker adhesion, CD56^+^ monocytes demonstrate superior migration compared with CD56^−^ monocytes, which together likely result in their enhanced ability to exit the vasculature and exert immune functions at target sites.

### Both In Vitro and In Vivo GC Treatment Endows Monocytes With Enriched CD56^+^ Monocytes and Immunomodulatory Functions

Previous studies indicate that DEX can induce an anti-inflammatory subset of monocytes.^27^^,^^38^ In line with this, we found that the proportion of CD56^+^ monocytes is elevated in patients with VKHS and further increased after GC therapy,^9^ and this finding can be recapitulated in in vitro DEX-stimulation experiments (see Fig. 1). These results thus suggest that CD56^+^ monocytes may be a GC-inducible, anti-inflammatory subpopulation involved in immune regulation in VKHS.

**Figure 6.**
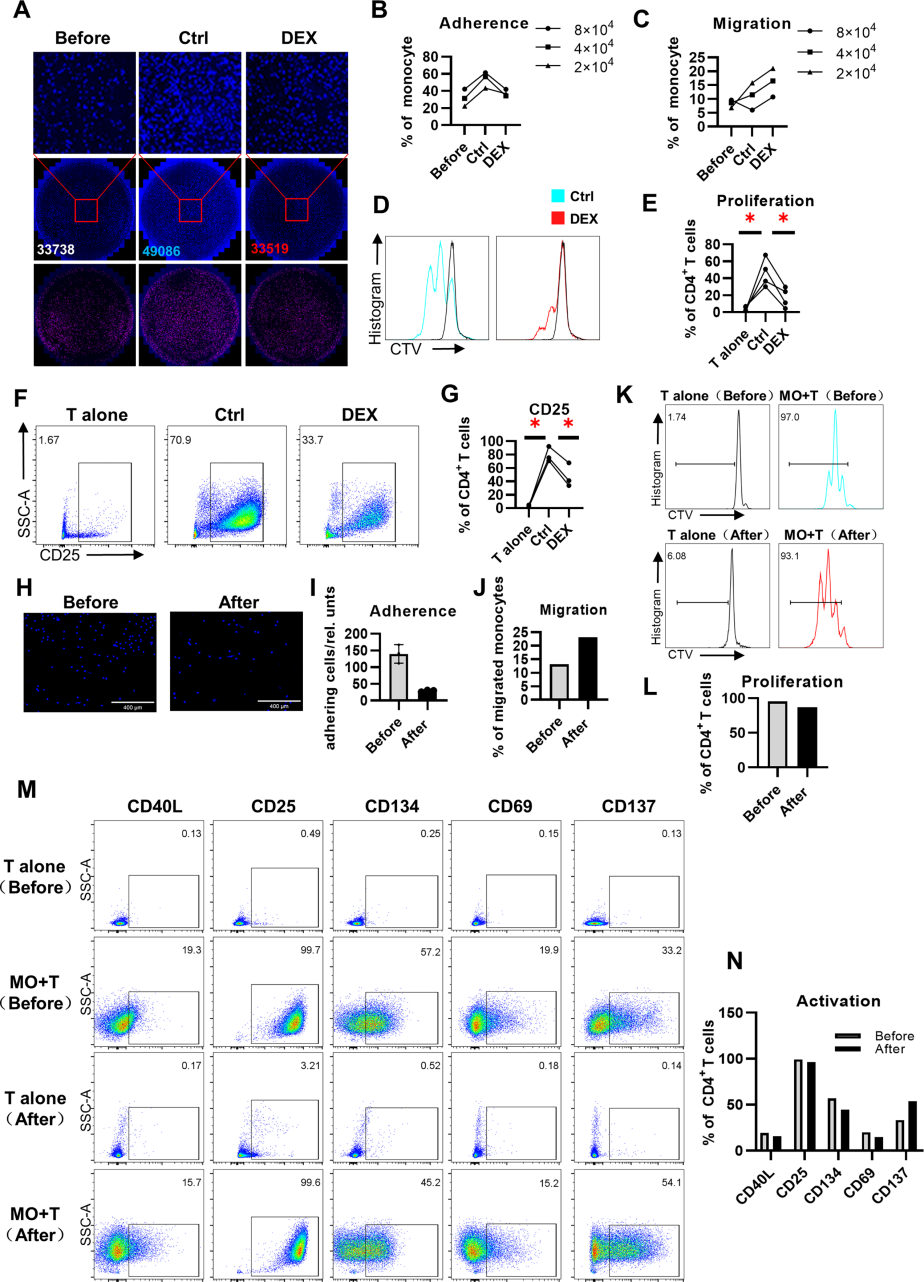
**In vitro and in vivo GC treatment enhances CD56^+^ monocyte abundance and immunomodulatory traits.** (**A**) DAPI fluorescence images of monocyte adhesion capacity captured by the EVOS M7000 multi-well plate scanning microscope. The first row shows a representative DAPI immunofluorescence staining image, which is the second row of selected area image magnification of 5×. The second row displays automated counting results from the EVOS M7000, and the third row represents DAPI signals scanned across an entire well in the 96-well plate. (**B**) Adhesion rates of monocytes were quantified based on DAPI staining. (**C**) Migration rates among the three groups were compared. (**D**) Monocytes, either stimulated with dexamethasone (DEX) or unstimulated (Ctrl), were co-cultured with CTV-labeled naïve CD4^+^ T cells in CD3-coated 96-well plates for 72 hours to evaluate CD4^+^ T cell proliferation. Histogram showing generations of CD4^+^ T cell proliferation revealed by CTV dilution. (**E**) Line graph depicting the percentage of proliferating CD4^+^ T cells and the statistical difference between indicated groups showed in **D** (*n* = 4). (**F**) Representative flow cytometric plots illustrating the expression of CD25, an activation marker, on CD4^+^ T cells at the same time point after co-culture as described in **D**. (**G**) Line graph showing the percentage of CD25^+^CD4^+^ T cells and the statistical difference between indicated groups showed in **D** (*n* = 3). (**H**) DAPI fluorescence images showing the adhering capacity of monocytes isolated from a patient with VKHS before and after GC treatment to plastic plates under serum-free medium. DAPI emits blue fluorescence; magnification: 200 ×. (**I**) Statistical comparison of the number of adhered cells calculated from **H**. Each of the three data points represents a technical replicate well. Each point indicates the average cell count across five randomly selected fields per well. (**J**) Migration rates among the two groups were compared. (**K**) Monocytes and CD4^+^ T cells isolated from a patient with VKHS before and after GC treatment were co-cultured in 96-well plates coated with an anti-CD3 antibody for 72 hours to assess CD4^+^ T cell proliferation. The histogram shows the generations of CD4^+^ T cell proliferation revealed by CTV dilution. (**L**) Statistical graph depicting the proportion of proliferating CD4^+^ T cells in indicated groups. (**M**) Flow cytometry plots showing the expression levels of activation markers on CD4^+^ T cells, including CD40L, CD25, CD134, CD69, and CD137. The experimental system is the same as **K**. (**N**) Histograms summarizing the percentages of activated CD4^+^ T cells expressing the markers indicated in **M**. DEX, dexamethasone; Ctrl, control; MO, monocyte; ***P < 0.001; *P < 0.05; NS, not significant. Datasets with three groups were analyzed using 1-way ANOVA, followed by Dunnett’s multiple comparisons test.

To test this premise, we first sought to determine whether in vitro DEX stimulation induces the functional changes of monocytes in addition to increasing the proportion of CD56^+^ monocytes. For this purpose, purified CD14^+^ monocytes were treated with either DEX or medium (control group) for 5 hours, and cell adhesion experiments were performed on these cells and those immediately after isolation (before group). As shown in Figures 6A and 6B, DEX stimulation significantly reduced the adhesion capacity of monocytes. Of note, the before group also had fewer adherent cells compared with controls, possibly due to isolation-related effects. Next, we performed Transwell assays to examine the three groups for their migrating capacity, revealing enhanced migrating capacity in DEX-stimulated monocytes (Fig. 6C). Thus, in line with the comparison between CD56^+^ monocytes and CD56^−^ monocytes, DEX-stimulated monocytes, which are enriched in CD56^+^ monocytes, exhibit lower adhesion and higher migrating capacities than untreated counterparts that are predominantly CD56^−^ monocytes.

We next asked if monocytes induced by DEX-stimulation have an immunoregulatory effect on CD4^+^ T cells, the primary immune cells mediating various autoimmune diseases.^39^ To test this, we separately isolated monocytes and naïve CD4^+^ T cells and co-cultured them for 72 hours after incubating the monocytes with DEX or without (Ctrl) DEX stimulation for 5 hours. The results showed that DEX-stimulated monocytes markedly suppressed the proliferation of naïve CD4^+^ T cells compared with controls (Figs. 6D, 6E). We also examined the activation markers of CD4^+^ T cells and found that the expression of CD25 on CD4^+^ T cells was significantly lower in the DEX-treated group compared to the control group (Figs. 6F, 6G), albeit other activation markers such as CD134 (OX40), CD137 (4-1BB), CD69, and CD154 (CD40L) did not show significant differences (Supplementary Figs. S4A–S4D). In summary, DEX stimulation induces changes in monocytes, enhancing their detachment and migration capabilities while suppressing CD4^+^ T cell proliferation and activation, highlighting CD56^+^ monocytes, which are enriched after DEX stimulation, as a potential anti-inflammatory subpopulation crucial for immune regulation.

Having found the phenotypical and functional changes of monocytes after in vitro DEX stimulation, we next sought to test if similar alterations can be observed in monocytes from patients with VKHS post-GC treatments. For this purpose, we collected peripheral blood from a patient with VKHS before and after GC therapy for 7 days. Indeed, monocytes from the patient with VKHS post-treatment also exhibited reduced adhesion and enhanced migration capacity (Figs. 6H–J), in addition to the elevated proportion of CD56^+^ monocytes. Importantly, when these monocytes were co-cultured with naïve CD4^+^ T cells, those after GC treatment showed reduced ability to stimulate CD4^+^ T cell proliferation (lower CTV^+^ cells) and weaker induction of CD4^+^ T cell activation (decreased CD25^+^, CD134^+^, and CD69^+^ cells) compared to the pretreatment group (Figs. 6K–N). Therefore, these results, together with the in vitro DEX stimulation experiments, indicates that in patients with VKHS post-GC therapy, monocytes are more likely to detach from the vasculature and migrate to specific sites to perform immunoregulatory function such as suppress CD4^+^ T cell proliferation and activation, thereby alleviating disease progression.

## Discussion

CD56 has been traditionally regarded as a lineage-specific marker of human NK cells. However, CD56 expressions on other cells of either myeloid or lymphoid lineages, including activated T cells, DCs, and monocytes, have also been reported. Specifically, Sconocchia et al. identified in healthy subjects a CD56-expressing cell population that co-expresses myeloid markers typically found on monocytes, such as CD14, CD33, CD11b, and HLA-DR, and therefore denoted this cell as CD33^+^CD56^low^ monocyte.^16^ However, whether this cell constitutes an independent monocyte subpopulation with unique phenotypic and functional characteristics remains obscure. This study, by performing comprehensively morphological, transcriptional, immunophenotypic, and functional comparisons of CD56^+^ monocytes, CD56^−^ monocytes, and NK cells, demonstrates that CD56^+^ monocyte is indeed a unique subset of monocytes exhibiting hybrid features of both monocytes and NK cells. On one hand, it displays a typical morphology of monocytes, expresses multiple monocyte markers at both transcriptional and protein levels, and can perform classic functions of monocytes including adhesion, migration, and antigen presentation, therefore confirming its identity as a bona fide monocyte subpopulation; on the other hand, in addition to CD56, this cell also expresses cytotoxicity-related molecules such as perforin and granzyme B, resembling NK cells but not CD56^−^ monocytes.

It has been widely accepted that human monocytes can be classified based on the expression of monocytic marker CD14 and another marker expressed on NK cells and neutrophils, CD16 (FcγRIII), into three subsets: classical monocyte (CD14^++^CD16^–^ or CD14^+^CD16^–^), intermediate monocyte (CD14^++^CD16^+^ or CD14^+^CD16^+^), and non-classical monocyte (CD14^+^CD16^++^ or CD14^dim^CD16^+^). However, the classification based on CD56 expression, as described by us and others, seems to be outside this paradigm: CD56^+^ monocytes and CD56^−^ monocytes harbor similar composition of classic, intermediate, and non-classic subpopulations (see Ref. 16 and data not shown). Nevertheless, this study provides compelling evidence showing the profound differences between CD56^+^ and CD56^−^ monocytes.

From the perspective of morphology, although previous studies observed that CD56^+^ monocytes exhibit glassy cytoplasm and large, irregular nuclei typical of monocytes,^16^ their difference between CD56^−^ monocytes has not been explored at the subcellular level, likely due to technical limitations. Our study, through combining confocal microscopy with flow cytometry analyses, is able to analyze the content and distribution of mitochondria and lysosome at the nanometer level, revealing that CD56^+^ monocytes contain more mitochondria, suggesting their distinct mitochondria-related functions. Mitochondria are one of the primary sites for the production of reactive oxygen species (ROS) within cells,^40^ whereas the balance between ROS production and clearance under physiological conditions is tightly regulated. Depending on the context, regulated oxidative stress can initiate various cellular responses, including triggering signaling pathways involved in cell protection, coordinating the activation of mitochondrial fission and autophagy, and clearing damaged mitochondria and cells to prevent the spread of injury to neighboring mitochondria and cells.^41^^,^^42^ Conversely, excessive oxidative and reductive stress can lead to severe cellular damage and unnecessary cell death, ultimately resulting in organ and organismal failure.^43^ In autoimmune disease models, it has been found that macrophages can induce the production of Tregs by secreting ROS, which suppresses T cell proliferation and induces T cell apoptosis.^44^ In our study, we found that CD56^+^ monocytes produce more ROS compared with CD56^−^ monocytes and NK cells, which is in line with their higher mitochondria content, suggesting that ROS may play a crucial role in maintaining the dynamic balance between protection and damage within this cell subset; however, this speculation warrants further investigation.

This study is the first to compare the transcriptional levels of functionally relevant molecules in CD56^+^ monocytes, CD56^−^ monocytes, and NK cells. We found that CD56^+^ monocytes express typical monocyte markers such as *CD14*, *CD33*, *CD11B*, *CD86*, *HLA-DRB1*, and *IL1B*, which is consistent with their protein expression levels. However, assessment on the protein expression levels of these molecules found that compared with CD56^−^ monocytes, CD56^+^ monocytes have significantly lower CD11b expression, indicative CD56^+^ monocytes of a less typical myeloid characteristic. Additionally, CD56^+^ monocytes express less adhesion molecule CD54 than CD56^−^ monocytes, whereas they express more activation/antigen-presenting molecules (HLA-DR and CD80), phagocytosis-related molecules (CD64 and CD163), and chemotaxis-related molecules (CX3CR1 and CCR5), suggesting that CD56^+^ monocytes are more prone to activation and migration to inflammatory sites. Interestingly, CD56^+^ monocytes also express NK cell marker genes including *NKp30*, *NKp46*, *NKG2D*, *GZMB*, and *PRF1*, which is reminiscent of a subset of NK-like monocytes characterized by the co-expression of both monocyte and NK cell marker genes (*CCL5*, *IL-32*, *GZMA*, *CST7*, *KLRB1*, *CD69*, *NKG7*, *GNLY*, *TRDC*, and *CCL4*), as identified by single-cell sequencing.^31^^,^^45^ However, unlike these studies, CD56^+^ monocytes in this study are distinguished by the protein expression of CD56, a membrane molecule that is also related to cell cytotoxicity, which facilitates their isolation and manipulation, providing a foundation for further functional exploration and the design of clinical intervention strategies targeting these cells. Of note, our RT-PCR results showed that CD56^+^ monocytes express higher levels of *NCAM1* (encodes CD56) compared with CD56^−^ monocytes, but *NCAM1* was not among the differentially expressed genes in transcriptomic sequencing. This discrepancy may be due to the low copy number of the CD56 gene, making it difficult to detect significant differences in transcriptomic sequencing. The difference in CD56 expression between transcriptomic sequencing and RT-PCR highlights the need for further validation of results obtained from RNA sequencing of small cell populations using RT-PCR and protein-level assays. For instance, in our study, CD56 protein expression was significantly higher in CD56^+^ monocytes compared with CD56^−^ monocytes. Together, our comparative analysis at the morphological, transcriptomic, and protein levels indicates that CD56^+^ monocytes represent an independent subset of cells with characteristics intermediate between CD56^−^ monocytes and NK cells, exhibiting both monocyte and NK cell phenotypes. These findings provide a basis for understanding the unique roles of CD56^+^ monocytes and their potential as therapeutic targets.

Monocytes can respond to inflammatory signals by secreting various cytokines. It has been reported that CD56^+^ monocytes can secrete certain levels of IL-6 and IL-1β.^16^ Additionally, studies have shown that CD56^+^ monocytes, when stimulated by LPS, secrete higher levels of TNF-α, IL-23, and IL-1β compared with CD56^−^ monocytes, suggesting a primarily pro-inflammatory role.^18^ Therefore, in this study, we used LPS stimulation to simulate an inflammatory environment and found that CD56^+^ monocytes secrete significantly higher proportions of pro-inflammatory cytokines such as TNF-α, IL-23, IL-8, and IL-6 compared with CD56^−^ monocytes and NK cells, consistent with previous findings. However, our study also revealed that CD56^+^ monocytes secrete higher levels of the anti-inflammatory cytokine IL-10 compared with CD56^−^ monocytes. These observations raise the question of whether CD56^+^ monocytes predominantly exert pro-inflammatory or anti-inflammatory effects, or if they play a dual role in maintaining a dynamic balance during inflammation. Although our results suggest that CD56^+^ monocytes are anti-inflammatory in VKHS (discuss later), further investigation is warranted to clarify the exact role of CD56^+^ monocytes in inflammation and how they contribute to achieving this balance.

Granzyme B and perforin are primarily expressed in cytotoxic T lymphocytes (CTLs) and NK cells.^46^ We have, for the first time, discovered that CD56^+^ monocytes can secrete measurable levels of granzyme B and perforin in the steady state, suggesting that CD56^+^ monocytes may possess certain cytotoxic or regulatory functions. In autoimmune diseases such as rheumatoid arthritis, Crohn’s disease, and multiple sclerosis, a type of GC-induced TolDCs has been identified.^47^^–^^49^ These TolDCs can be generated by in vitro treatment of monocytes with GC.^33^^,^^34^ Additionally, perforin^+^ DCs, a subset of TolDCs, can limit autoreactive T cells and promote immune tolerance.^36^^,^^37^^,^^50^ It has also been observed that CD56^+^ DCs secrete more granzyme B compared with CD56^−^ DCs.^35^ These findings, together with the expression of granzyme B and perforin in CD56^+^ monocytes, suggest that CD56^+^ monocytes may be regulatory monocytes in human peripheral blood similar to TolDCs. Given that peripheral CD56^+^ monocytes in patients with VKHS are more abundant than in healthy subjects, and are further increased after GC therapy, it is tentative to speculate that CD56^+^ monocytes may represent a regulatory monocyte subpopulation responding to GC therapy in VKHS.

Consistent with this hypothesis, previous investigations reported that GC can extend the survival of monocytes and induce a specific anti-inflammatory monocyte phenotype capable of actively promoting inflammation resolution.^26^^,^^27^ This effect is unique and cannot be recapitulated by the addition of individual cytokines such as IL-4, IL-6, and IL-10.^27^ In line with these findings, our in vitro functional experiments also demonstrated that GC treatment enhances the phagocytic and migratory abilities of monocytes while reducing their adhesion capacity. This suggests that GC-treated monocytes are more likely to detach from blood vessels and migrate into tissues to exert their immune functions. Furthermore, GC-stimulated monocytes can inhibit the proliferation and activation of CD4^+^ T cells, which are considered the primary adaptive autoreactive cells targeting melanocytes in VKHS. Therefore, our results suggest that GC-induced CD56^+^ monocytes may not only regulate innate inflammatory responses but also indirectly modulate the onset and progression of VKHS by inhibiting CD4^+^ T cells, thereby mediating patients’ responsiveness to GC treatment. Consistent with this speculation, our study provides the first, to the best of our knowledge, evidence that in patients with VKHS treated with GCs, monocytes exhibit reduced adhesion and enhanced migration, and effectively inhibit the proliferation and activation of CD4^+^ T cells. Considering the similar properties of CD56^+^ monocyte, and the increased proportion of this cell population after GC therapy, it is very likely that as a regulatory monocyte in patients with VKHS, CD56^+^ monocytes can be further induced by GC therapy, and subsequently migrate to inflammatory sites to suppress CD4^+^ T cell proliferation and activation, thus limiting damage to melanocytes.

This study has come with some limitations. First, we examined peripheral blood instead of aqueous humor (AH) that is known for its ocular specificity, for the alterations of monocytes. Indeed, AH is capable of faithfully reflecting ocular inflammation and patient's response to therapy, making it an ideal site for investigating ocular inflammatory diseases including VKHS.^51^^,^^52^ We failed to examine what happens in the AH is largely due to the more limited availability of AH than periphery blood in the real-world clinical practice. Nevertheless, it would be very interesting to investigate the cellular and molecular events in AH in our future study. Second, some therapeutic regimens, for example, intraocular injection of drugs including corticosteroids and systemic application of drugs other than corticosteroids, have demonstrated efficacy in some patients with VKHS for whom the standard corticosteroid therapeutic regimen is not applicable.^53^^–^^62^ However, we were not able to investigate the alteration of monocytes under these conditions, which therefore warrants further exploration.

In summary, our study demonstrates from transcriptomic, protein, and morphological perspectives that CD56^+^ monocytes function as an independent monocyte subset. The increased abundance of CD56^+^ monocytes in VKHS following GC treatment, coupled with their reduced adhesion capability, and increased capacity to migrating into inflamed sites and suppressing CD4^+^ T cells, together suggest that these cells might be a population of regulatory monocyte induced by GC therapy to reduce damage to melanocytes and alleviate inflammation. Our findings and subsequent in-depth mechanistic investigations will lay the foundation for understanding the pathogenesis of VKHS and the mechanisms underlying patients’ responsiveness to GC treatment. Additionally, they provide a basis for developing novel therapeutic strategies through harnessing CD56^+^ monocytes for the treatment of VKHS.

## Supplementary Material

Supplement 1

## References

[1] Moorthy RS, Inomata H, Rao NA. Vogt-Koyanagi-Harada syndrome. *Surv Ophthalmol*. 1995; 39(4): 265–292. 7725227 10.1016/s0039-6257(05)80105-5

[2] Lavezzo MM, Sakata VM, Morita C, et al. Vogt-Koyanagi-Harada disease: review of a rare autoimmune disease targeting antigens of melanocytes. *Orphanet J Rare Dis*. 2016; 11: 29. 27008848 10.1186/s13023-016-0412-4 PMC4806431

[3] Zhao M, Jiang Y, Abrahams IW. Association of HLA antigens with Vogt-Koyanagi-Harada syndrome in a Han Chinese population. *Arch Ophthalmol*. 1991; 109(3): 368–370. 2003797 10.1001/archopht.1991.01080030070041

[4] Davis JL, Mittal KK, Freidlin V, et al. HLA associations and ancestry in Vogt-Koyanagi-Harada disease and sympathetic ophthalmia. *Ophthalmology*. 1990; 97(9): 1137–1142. 2234843 10.1016/s0161-6420(90)32446-6

[5] Islam SM, Numaga J, Fujino Y, et al. HLA class II genes in Vogt-Koyanagi-Harada disease. *Invest Ophthalmol Vis Sci*. 1994; 35(11): 3890–3896. 7928186

[6] Sugita S, Takase H, Kawaguchi T, Taguchi C, Mochizuki M. Cross-reaction between tyrosinase peptides and cytomegalovirus antigen by T cells from patients with Vogt-Koyanagi-Harada disease. *Int Ophthalmol*. 2007; 27(2–3): 87–95. 17253112 10.1007/s10792-006-9020-y

[7] Damico FM, Cunha-Neto E, Goldberg AC, et al. T-cell recognition and cytokine profile induced by melanocyte epitopes in patients with HLA-DRB1*0405-positive and -negative Vogt-Koyanagi-Harada uveitis. *Invest Ophthalmol Vis Sci*. 2005; 46(7): 2465–2471. 15980237 10.1167/iovs.04-1273

[8] Maezawa N, Yano A. Two distinct cytotoxic T lymphocyte subpopulations in patients with Vogt-Koyanagi-Harada disease that recognize human melanoma cells. *Microbiol Immunol*. 1984; 28(2): 219–231. 6203018 10.1111/j.1348-0421.1984.tb00673.x

[9] Jiang H, Li Z, Yu L, et al. Immune phenotyping of patients with acute Vogt-Koyanagi-Harada syndrome before and after glucocorticoids therapy. *Front Immunol*. 2021; 12: 659150. 33995378 10.3389/fimmu.2021.659150 PMC8113950

[10] Jakubzick CV, Randolph GJ, Henson PM. Monocyte differentiation and antigen-presenting functions. *Nat Rev Immunol*. 2017; 17(6): 349–362. 28436425 10.1038/nri.2017.28

[11] Rigamonti A, Villar J, Segura E. Monocyte differentiation within tissues: a renewed outlook. *Trends Immunol*. 2023; 44(12): 999–1013. 37949783 10.1016/j.it.2023.10.005

[12] Shi C, Pamer EG. Monocyte recruitment during infection and inflammation. *Nat Rev Immunol*. 2011; 11(11): 762–774. 21984070 10.1038/nri3070 PMC3947780

[13] Orozco SL, Canny SP, Hamerman JA. Signals governing monocyte differentiation during inflammation. *Curr Opin Immunol*. 2021; 73: 16–24. 34411882 10.1016/j.coi.2021.07.007 PMC8648978

[14] van Furth R. Monocyte production during inflammation. *Comp Immunol Microbiol Infect Dis*. 1985; 8(2): 205–211. 3910343 10.1016/0147-9571(85)90045-1

[15] Feldmann M, Brennan FM, Maini RN. Role of cytokines in rheumatoid arthritis. *Ann Rev Immunol*. 1996; 14: 397–440. 8717520 10.1146/annurev.immunol.14.1.397

[16] Sconocchia G, Keyvanfar K, El Ouriaghli F, et al. Phenotype and function of a CD56+ peripheral blood monocyte. *Leukemia*. 2005; 19(1): 69–76. 15526027 10.1038/sj.leu.2403550

[17] Grip O, Bredberg A, Lindgren S, Henriksson G. Increased subpopulations of CD16(+) and CD56(+) blood monocytes in patients with active Crohn's disease. *Inflamm Bowel Dis*. 2007; 13(5): 566–572. 17260384 10.1002/ibd.20025

[18] Krasselt M, Baerwald C, Wagner U, Rossol M. CD56+ monocytes have a dysregulated cytokine response to lipopolysaccharide and accumulate in rheumatoid arthritis and immunosenescence. *Arthritis Res Ther*. 2013; 15(5): R139. 24286519 10.1186/ar4321 PMC3978677

[19] Franco LM, Gadkari M, Howe KN, et al. Immune regulation by glucocorticoids can be linked to cell type-dependent transcriptional responses. *J Exp Med*. 2019; 216(2): 384–406. 30674564 10.1084/jem.20180595 PMC6363437

[20] Liberman AC, Budziñski ML, Sokn C, Gobbini RP, Steininger A, Arzt E. Regulatory and mechanistic actions of glucocorticoids on T and inflammatory cells. *Front Endocrinol (Lausanne)*. 2018; 9: 235. 29867767 10.3389/fendo.2018.00235 PMC5964134

[21] Cain DW, Cidlowski JA. Immune regulation by glucocorticoids. *Nat Rev Immunol*. 2017; 17(4): 233–247. 28192415 10.1038/nri.2017.1 PMC9761406

[22] Di Rosa M, Radomski M, Carnuccio R, Moncada S. Glucocorticoids inhibit the induction of nitric oxide synthase in macrophages. *Biochem Biophys Res Commun*. 1990; 172(3): 1246–1252. 1700905 10.1016/0006-291x(90)91583-e

[23] Lee SH, Soyoola E, Chanmugam P, et al. Selective expression of mitogen-inducible cyclooxygenase in macrophages stimulated with lipopolysaccharide. *J Biol Chem*. 1992; 267(36): 25934–25938. 1464605

[24] Mozo L, Suárez A, Gutiérrez C. Glucocorticoids up-regulate constitutive interleukin-10 production by human monocytes. *Clin Exp Allergy*. 2004; 34(3): 406–412. 15005734 10.1111/j.1365-2222.2004.01824.x

[25] Högger P, Dreier J, Droste A, Buck F, Sorg C. Identification of the integral membrane protein RM3/1 on human monocytes as a glucocorticoid-inducible member of the scavenger receptor cysteine-rich family (CD163). *J Immunol*. 1998; 161(4): 1883–1890. 9712057

[26] Ehrchen J, Steinmüller L, Barczyk K, et al. Glucocorticoids induce differentiation of a specifically activated, anti-inflammatory subtype of human monocytes. *Blood*. 2007; 109(3): 1265–1274. 17018861 10.1182/blood-2006-02-001115

[27] Tsianakas A, Varga G, Barczyk K, et al. Induction of an anti-inflammatory human monocyte subtype is a unique property of glucocorticoids, but can be modified by IL-6 and IL-10. *Immunobiology*. 2012; 217(3): 329–335. 22154546 10.1016/j.imbio.2011.10.002

[28] Girndt M, Sester U, Kaul H, Hünger F, Köhler H. Glucocorticoids inhibit activation-dependent expression of costimulatory molecule B7-1 in human monocytes. *Transplantation*. 1998; 66(3): 370–375. 9721807 10.1097/00007890-199808150-00015

[29] Varga G, Ehrchen J, Brockhausen A, et al. Immune suppression via glucocorticoid-stimulated monocytes: a novel mechanism to cope with inflammation. *J Immunol*. 2014; 193(3): 1090–1099. 24990080 10.4049/jimmunol.1300891

[30] Dan Dunn J, Alvarez LA, Zhang X, Soldati T. Reactive oxygen species and mitochondria: a nexus of cellular homeostasis. *Redox Biol*. 2015; 6: 472–485. 26432659 10.1016/j.redox.2015.09.005 PMC4596921

[31] Hu Y, Hu Y, Xiao Y, et al. Genetic landscape and autoimmunity of monocytes in developing Vogt-Koyanagi-Harada disease. *Proc Natl Acade Sci USA*. 2020; 117(41): 25712–25721. 10.1073/pnas.2002476117 PMC7568339 32989127

[32] Prager I, Watzl C. Mechanisms of natural killer cell-mediated cellular cytotoxicity. *J Leukoc Biol*. 2019; 105(6): 1319–1329. 31107565 10.1002/JLB.MR0718-269R

[33] Xia CQ, Peng R, Beato F, Clare-Salzler MJ. Dexamethasone induces IL-10-producing monocyte-derived dendritic cells with durable immaturity. *Scand J Immunol*. 2005; 62(1): 45–54. 16091124 10.1111/j.1365-3083.2005.01640.x

[34] Naranjo-Gómez M, Raïch-Regué D, Oñate C, et al. Comparative study of clinical grade human tolerogenic dendritic cells. *J Transl Med*. 2011; 9: 89. 21658226 10.1186/1479-5876-9-89 PMC3141500

[35] Anguille S, Lion E, Tel J, et al. Interleukin-15-induced CD56(+) myeloid dendritic cells combine potent tumor antigen presentation with direct tumoricidal potential. *PLoS One*. 2012; 7(12): e51851. 23284789 10.1371/journal.pone.0051851 PMC3532168

[36] Zlotnikov-Klionsky Y, Nathansohn-Levi B, Shezen E, et al. Perforin-positive dendritic cells exhibit an immuno-regulatory role in metabolic syndrome and autoimmunity. *Immunity*. 2015; 43(4): 776–787. 26384546 10.1016/j.immuni.2015.08.015

[37] Zangi L, Klionsky YZ, Yarimi L, et al. Deletion of cognate CD8 T cells by immature dendritic cells: a novel role for perforin, granzyme A, TREM-1, and TLR7. *Blood*. 2012; 120(8): 1647–1657. 22776817 10.1182/blood-2012-02-410803

[38] Ehrchen JM, Roth J, Barczyk-Kahlert K. More than suppression: glucocorticoid action on monocytes and macrophages. *Front Immunol*. 2019; 10: 2028. 31507614 10.3389/fimmu.2019.02028 PMC6718555

[39] Krovi SH, Kuchroo VK. Activation pathways that drive CD4(+) T cells to break tolerance in autoimmune diseases. *Immunol Rev*. 2022; 307(1): 161–190. 35142369 10.1111/imr.13071 PMC9255211

[40] Kausar S, Wang F, Cui H. The role of mitochondria in reactive oxygen species generation and its implications for neurodegenerative diseases. *Cells*. 2018; 7(12): 274. 30563029 10.3390/cells7120274 PMC6316843

[41] Hoye AT, Davoren JE, Wipf P, Fink MP, Kagan VE. Targeting mitochondria. *Acc Chem Res*. 2008; 41(1): 87–97. 18193822 10.1021/ar700135m

[42] Zorov DB, Bannikova SY, Belousov VV, et al. Reactive oxygen and nitrogen species: friends or foes? *Biochemistry (Mosc)*. 2005; 70(2): 215–221. 15807661 10.1007/s10541-005-0103-6

[43] Zorov DB, Plotnikov EY, Jankauskas SS, et al. The phenoptosis problem: what is causing the death of an organism? Lessons from acute kidney injury. *Biochemistry (Mosc)*. 2012; 77(7): 742–753. 22817538 10.1134/S0006297912070073

[44] Ohl K, Tenbrock K, Kipp M. Oxidative stress in multiple sclerosis: central and peripheral mode of action. *Exp Neurol*. 2016; 277: 58–67. 26626971 10.1016/j.expneurol.2015.11.010 PMC7094520

[45] Yao J, Liu T, Zhao Q, et al. Genetic landscape and immune mechanism of monocytes associated with the progression of acute-on-chronic liver failure. *Hepatol Int*. 2023; 17(3): 676–688. 36626090 10.1007/s12072-022-10472-y PMC10224851

[46] Waterhouse NJ, Trapani JA. CTL: caspases terminate life, but that's not the whole story. *Tissue Antigens*. 2002; 59(3): 175–183. 12074707 10.1034/j.1399-0039.2002.590301.x

[47] Bell GM, Anderson AE, Diboll J, et al. Autologous tolerogenic dendritic cells for rheumatoid and inflammatory arthritis. *Ann Rheum Dis*. 2017; 76(1): 227–234. 27117700 10.1136/annrheumdis-2015-208456 PMC5264217

[48] Jauregui-Amezaga A, Cabezón R, Ramírez-Morros A, et al. Intraperitoneal administration of autologous tolerogenic dendritic cells for refractory Crohn's disease: a phase I study. *J Crohns Colitis*. 2015; 9(12): 1071–1078. 26303633 10.1093/ecco-jcc/jjv144

[49] Phillips BE, Garciafigueroa Y, Trucco M, Giannoukakis N. Clinical tolerogenic dendritic cells: exploring therapeutic impact on human autoimmune disease. *Front Immunol*. 2017; 8: 1279. 29075262 10.3389/fimmu.2017.01279 PMC5643419

[50] Takenaka MC, Quintana FJ. Achieving tolerance with perforin-secreting dendritic cells. *Trends Mol Med*. 2016; 22(1): 3–4. 26700492 10.1016/j.molmed.2015.11.008 PMC4707090

[51] Bonacini M, Soriano A, Cimino L, et al. Cytokine profiling in aqueous humor samples from patients with non-infectious uveitis associated with systemic inflammatory diseases. *Front Immunol*. 2020; 11: 358. 32210963 10.3389/fimmu.2020.00358 PMC7077343

[52] Sakaguchi M, Sugita S, Sagawa K, Itoh K, Mochizuki M. Cytokine production by T cells infiltrating in the eye of uveitis patients. *Jpn J Ophthalmol*. 1998; 42(4): 262–268. 9749865 10.1016/s0021-5155(98)00016-1

[53] Tsubota K, Goto H, Asakage M, et al. Comparative study of efficacy and safety of pulse versus half-pulse steroid therapy for Vogt-Koyanagi-Harada Disease. *Jpn J Ophthalmol*. 2025; 69(5): 805–812. 40418489 10.1007/s10384-025-01213-3 PMC12391172

[54] Kurt ZE, Argin MA. Acute phase Vogt-Koyanagi-Harada syndrome resistant to corticosteroid therapy in an adult female patient. *Arch Soc Esp Oftalmol (Engl Ed)*. 2025; 100(11): 723–727. 40780442 10.1016/j.oftale.2025.08.003

[55] Sundararaju U, Subramanian S, Rajakumar HK. Steroid pulse therapy for VKH during pregnancy: a safe and effective option? *Orphanet J Rare Dis*. 2025; 20(1): 366. 40671112 10.1186/s13023-025-03916-9 PMC12265270

[56] Abe S, Nakamura T, Okumura E, Oiwake T, Okada AA, Hayashi A. Long-term changes of choroidal blood flow velocity in Vogt-Koyanagi-Harada disease. *Graefes Arch Clin Exp Ophthalmol*. 2022; 260(6): 1933–1939. 34982220 10.1007/s00417-021-05540-2

[57] Nakamura T, Keino H, Okada AA. Sub-tenon triamcinolone acetonide injection in a pregnant patient with Vogt-Koyanagi-Harada disease. *Retin Cases Brief Rep*. 2018; 12(4): 375–378. 28033228 10.1097/ICB.0000000000000510

[58] Reibaldi M, Russo A, Avitabile T, et al. Treatment of persistent serous retinal detachment in Vogt-Koyanagi-Harada syndrome with intravitreal bevacizumab during the systemic steroid treatment. *Retina (Philadelphia, Pa)*. 2014; 34(3): 490–496. 23903795 10.1097/IAE.0b013e3182a0e446

[59] Tabl AA, Elsayed MA, Tabl MA. Suprachoroidal triamcinolone acetonide injection: a novel therapy for serous retinal detachment due to Vogt-Koyanagi Harada disease. *Eur J Ophthalmol*. 2022; 32(6): 3482–3488. 35266801 10.1177/11206721221085420

[60] Cohen D, Ben-Arie-Weintrob Y, Hareuveni-Blum T, Naaman E. [Vogt-Koyanagi-Harada syndrome] [in Hebrew]. *Harefuah*. 2025; 164(3): 183–187. 40134159

[61] Zmuda M, Tiev KP, Knoeri J, Héron E. Successful use of infliximab therapy in sight-threatening corticosteroid-resistant Vogt-Koyanagi-Harada disease. *Ocul Immunol Inflamm*. 2013; 21(4): 310–316. 23617262 10.3109/09273948.2013.775312

[62] Nakai S, Takeuchi M, Usui Y, et al. Efficacy and safety of adalimumab for exacerbation or relapse of ocular inflammation in patients with Vogt-Koyanagi-Harada disease: a multicenter study. *Ocul Immunol Inflamm*. 2024; 32(4): 367–375. 35748779 10.1080/09273948.2022.2092007

