# Supplementary material for: CD14+CD56+ Cell Is an Independent Regulatory Monocyte Subpopulation Increased in VKHS Patients Following Glucocorticoids Therapy
Source: Invest Ophthalmol Vis Sci. 2026 Jul 24;67(8):53. doi: 10.1167/iovs.67.8.53 (PMC13421833; doi:10.1167/iovs.67.8.53)
Supplement: Supplement 1 [file iovs-67-8-53_s001.docx]

**Supplementary Information**

**CD14^+^CD56^+^ cell is an independent regulatory monocyte subpopulation increased in VKHS patients following glucocorticoids therapy**

**This file includes 4 supplementary figures:**


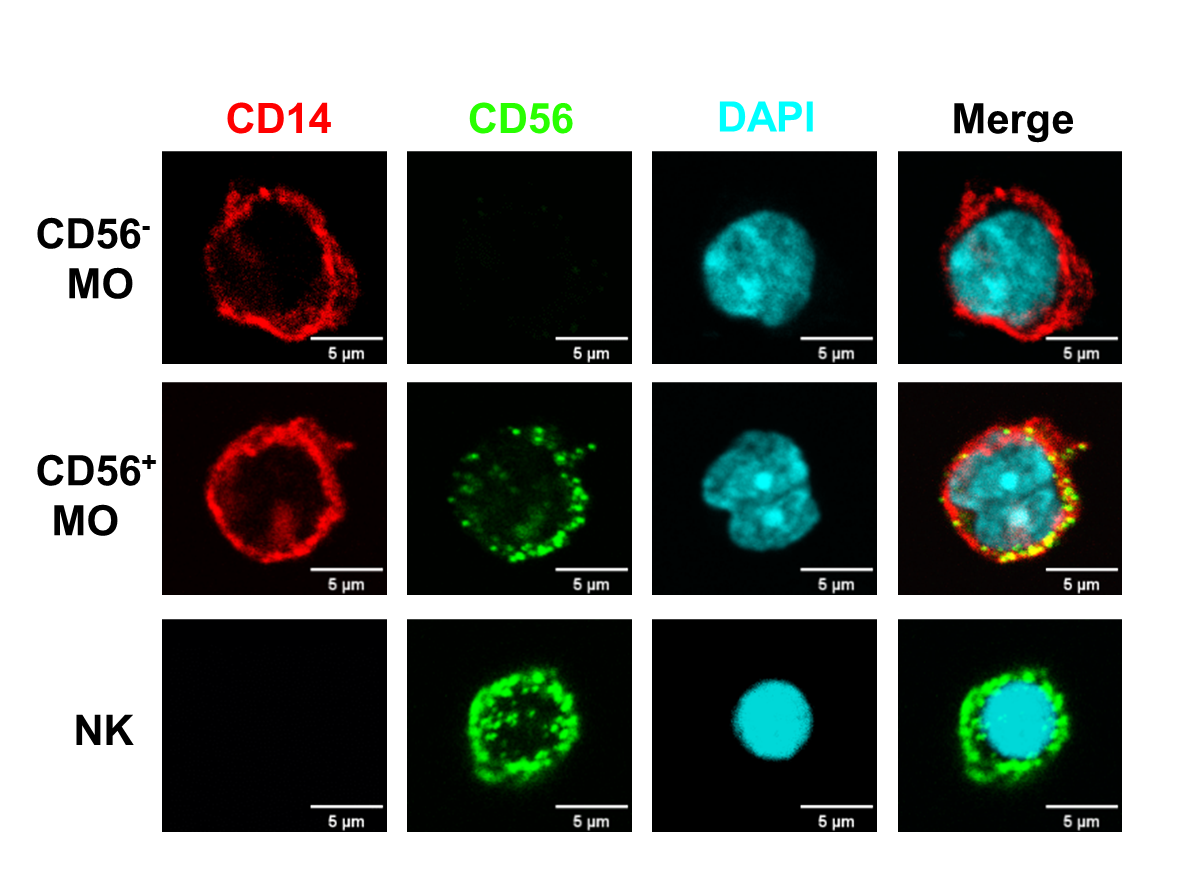


**Fig.S1.** Representative confocal laser microscopy images of in CD56^+^ monocytes, CD56^−^ monocytes, and NK cells. CD14 is labeled in red, CD56 in green, and DAPI in cyan. MO, monocytes.


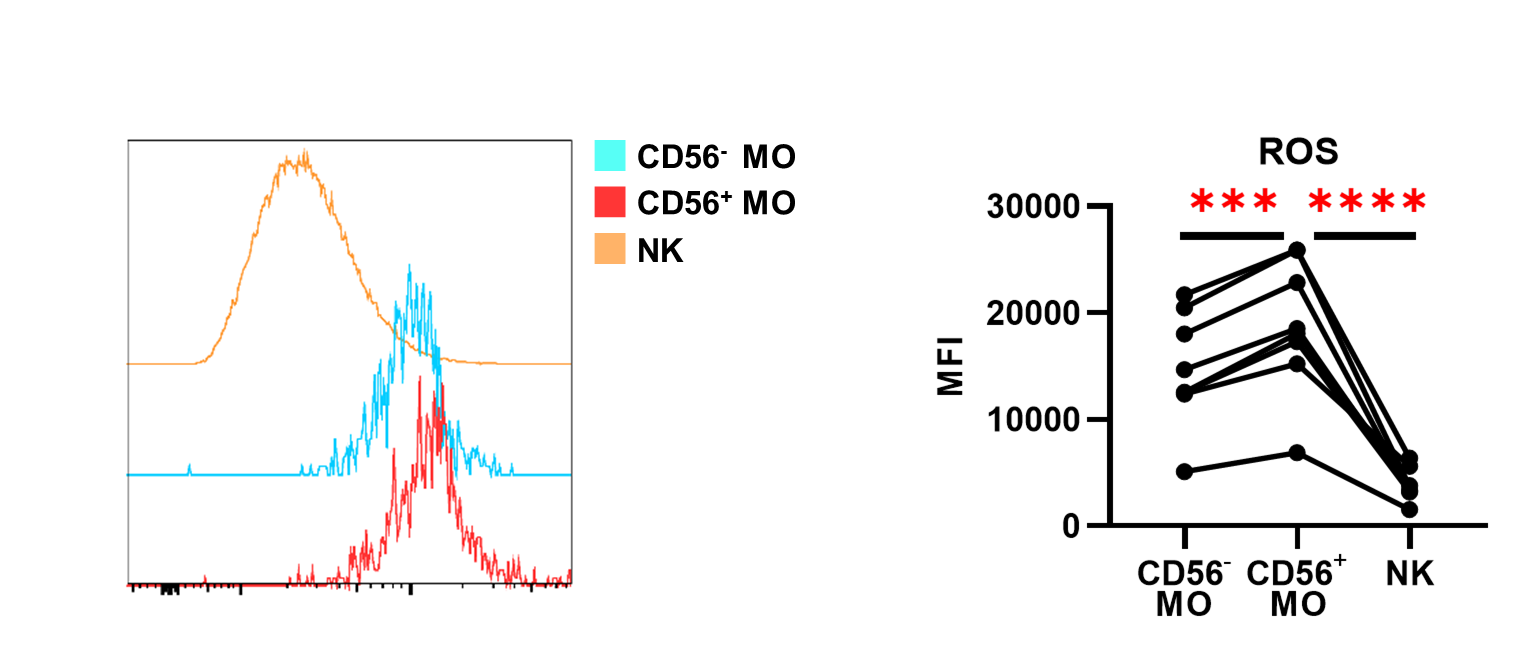


**Fig.S2.** ROS levels in CD14^+^CD56^-^ monocytes, CD14^+^CD56^+^monocytes, and CD14^-^CD56^+^ NK cells were detected by flow cytometry (n = 8). MO, monocytes; *****P* < 0.0001; ****P* < 0.001. Datasets with three groups were analyzed using one-way ANOVA, followed by Dunnett's multiple comparisons test.


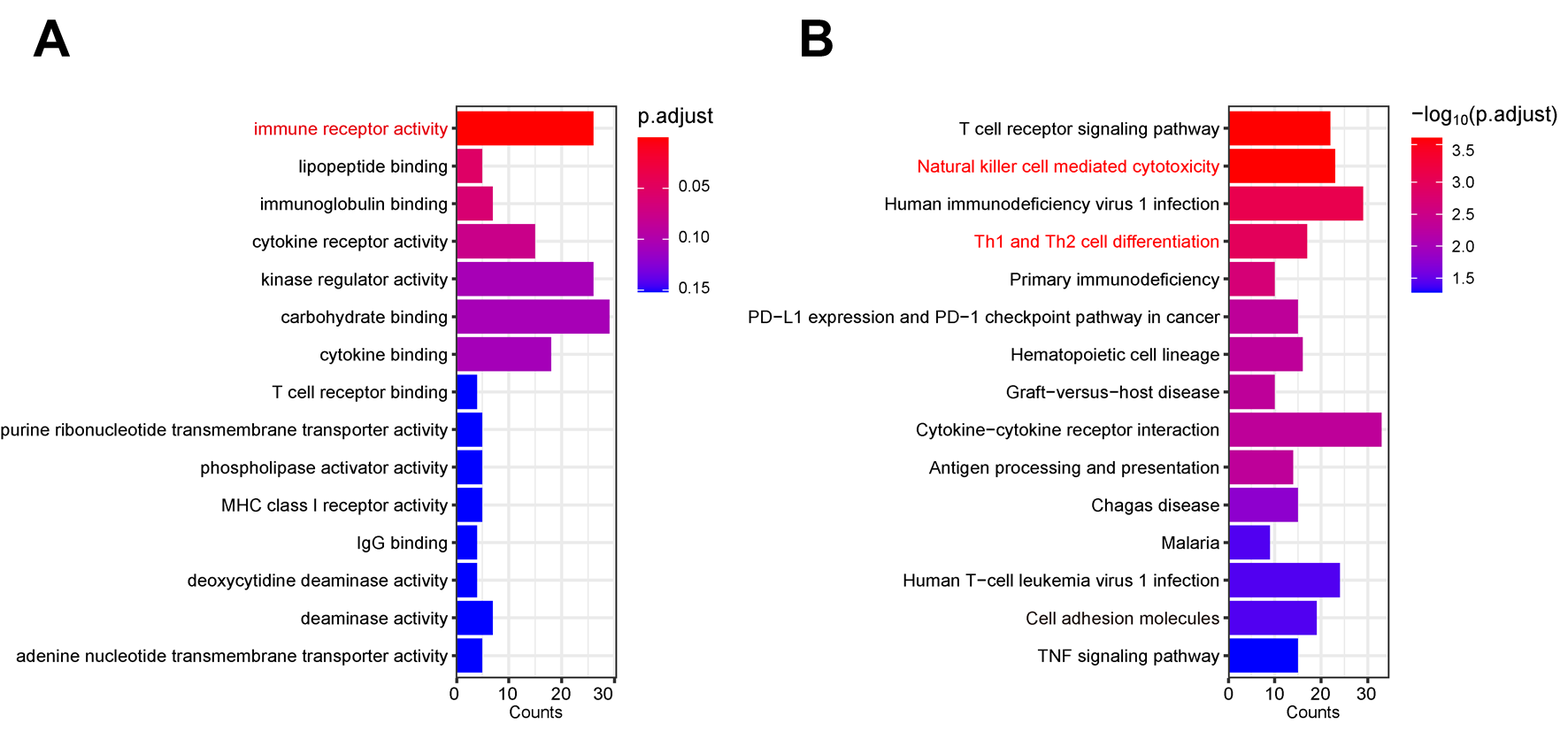


**Fig. S3.** (A) GO analysis results for Molecular Function (MF). (B) KEGG pathway analysis results.


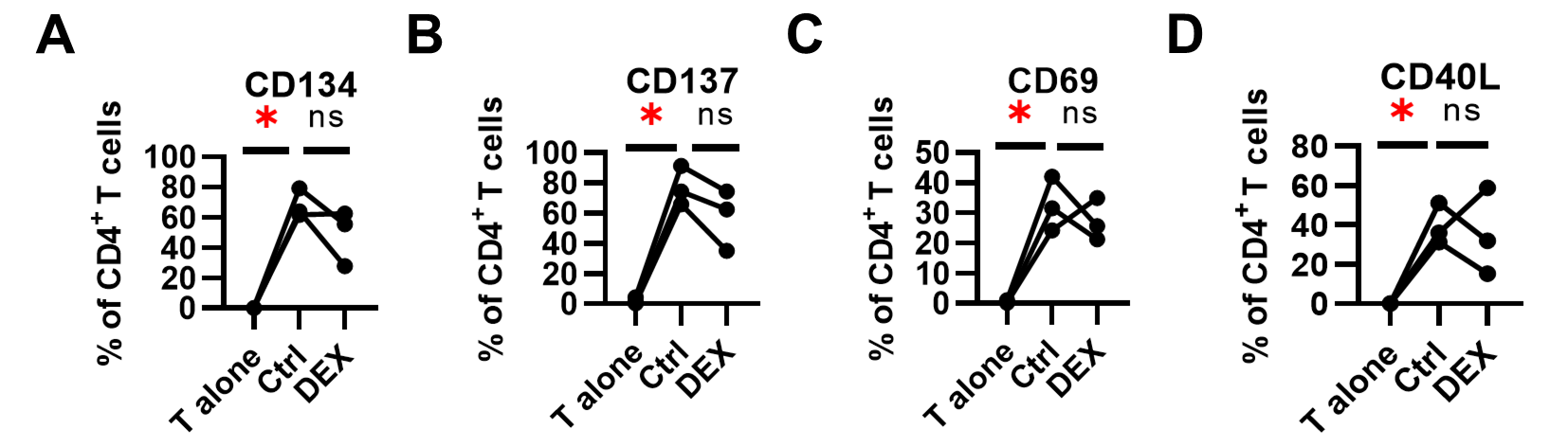


**Fig. S4.** (A–D) Line graph showing the statistical difference between indicated 3 groups in the percentages of CD4^+^ T cells expressing the activation markers CD134 (A), CD137 (B), CD69 (C), and CD40L (D) (n = 3). DEX, dexamethasone; Ctrl, control; MO, monocyte; ****P* < 0.001; **P* < 0.05; NS, not significant. Datasets with three groups were analyzed using one-way ANOVA, followed by Dunnett's multiple comparisons test.
